# ADAM10‐Mediated Proteolytic Remodelling of Signalling and Adhesion Proteins on Brain Cell‐Derived Small Extracellular Vesicles

**DOI:** 10.1002/jex2.70129

**Published:** 2026-04-17

**Authors:** Christopher C. Reimann, Hermann C. Altmeppen, Tomas Koudelka, Michaela Schweizer, Andreas Tholey, Behnam Mohammadi, Julia Bär, Lesley Cheng, Markus Glatzel, Marina Mikhaylova, Andrew F. Hill

**Affiliations:** ^1^ Department of Biochemistry and Chemistry, La Trobe Institute for Molecular Science La Trobe University Bundoora Victoria Australia; ^2^ Guest Group ‘Neuronal Protein Transport’, Center for Molecular Neurobiology University Medical Center Hamburg‐Eppendorf (UKE) Hamburg Germany; ^3^ Institute of Neuropathology University Medical Center Hamburg‐Eppendorf (UKE) Hamburg Germany; ^4^ Institute of Experimental Medicine Christian‐Albrechts‐Universität zu Kiel Kiel Germany; ^5^ Core Facility Morphology and Electron Microscopy, Center for Molecular Neurobiology University Medical Center Hamburg‐Eppendorf (UKE) Hamburg Germany; ^6^ AG Optobiology, Institute of Biology Humboldt‐Universität zu Berlin Berlin Germany; ^7^ Institute for Health and Sport Victoria University Melbourne Australia

**Keywords:** ADAM10, ectodomain shedding, extracellular vesicles, intercellular communication, neurobiology, prion protein, proteomics

## Abstract

Proteases are common components of extracellular vesicles (EVs), yet the extent and functional relevance of ongoing proteolytic activity on EV surfaces remain largely unexplored. Such activity could significantly influence EV function and identity, with likely implications for EV‐mediated signalling, recipient cell targeting, cargo delivery, and even translational applications ranging from biomarker discovery to therapeutic approaches. Here, we investigated the impact of sustained proteolysis on the composition of brain cell‐derived EVs, focusing on A Disintegrin And Metalloprotease 10 (ADAM10), a key sheddase for signalling and adhesion proteins involved in neuronal and synaptic processes. Using primary rat cortical cultures, we found that numerous known ADAM10 substrates are part of small EVs (sEVs), and that their associated functions overlap with major sEV‐mediated roles such as nervous system development, cell adhesion, and neurite outgrowth. Applying N‐terminal proteomics to monitor sEV‐derived cleavage fragments over time, we identified novel substrate candidates and demonstrated that sEV‐associated ADAM10 activity remodels surface proteins involved in EV‐cell interactions while generating soluble factors implicated in neuronal development. These findings suggest a previously unrecognised role for ADAM10 as a modulator of sEV composition and potentially cell‐targeting specificity in the brain and position EVs as dynamic platforms for proteolytic processing ‘on the move’.

## 1 Introduction

Proteases play pivotal roles in tissue development, remodelling, and cell‐cell communication by regulating the abundance and function of membrane proteins (e.g., adhesion proteins and receptors) and by releasing signalling‐competent ligands. Proteolytic cleavage of extracellular protein domains at the cell surface, referred to as ectodomain shedding, typically generates a membrane‐bound fragment and a soluble (‘shed’) fragment from a given substrate that may both exert functions different from the full‐length protein (comprehensively reviewed in Lichtenthaler et al., 2018). The ectodomain that is released into the extracellular space often acts as a potent biological modifier in an auto‐, para‐, or even endocrine manner. One of the best characterised sheddases is A Disintegrin And Metalloprotease 10 (ADAM10), a ubiquitously expressed and constitutively active type I transmembrane protease with an extracellular zinc‐dependent catalytic domain. With over 100 substrates ranging from cell adhesion molecules, signalling receptors, inflammatory mediators, and growth factors, ADAM10 modulates adhesive, attractive, and repulsive responses and plays crucial roles in development, immunological processes, cardiovascular diseases, and various types of cancer (for an overview, see Rosenbaum and Saftig, 2023).

In the brain, where neurons and glia cells rely on surface proteins for ligand‐receptor interactions and synaptic connectivity, as well as for growth and maintenance of their cellular projections, ADAM10 is a key player in neurodevelopment, synaptic plasticity, and neurite outgrowth (Hsia et al., 2019). Conversely, dysregulation of ADAM10 expression and/or activity has been implicated in a range of brain disorders, including Fragile X syndrome, autism, epilepsy, tumours, and different neurodegenerative diseases (Saftig and Lichtenthaler, 2015). Notably, in conditions such as Alzheimer's disease and prion diseases, ADAM10's proteolytic activity has been predominantly associated with neuroprotective effects (Altmeppen et al., 2015; Kuhn et al., 2010).

While ADAM10 is commonly present at the plasma membrane or in intracellular compartments (Bär et al., 2024; Gutwein et al., 2003; Lammich et al., 1999; Lundgren et al., 2015), it is also found as a frequent component of extracellular vesicles (EVs). Sharing the topology of their cell of origin, these lipid bilayer‐enclosed particles present ligands and receptors on their surface and contain cytoplasmic proteins and nucleic acids within their lumen, enabling them to shuttle biomolecular cargo (and thus biological information) across both short and long distances through the extracellular space and body fluids (Maas et al., 2017). As such, EVs have emerged as an important and increasingly prominent system for cell‐cell communication (Van Niel et al., 2018). Like transmembrane proteases, EVs are involved in key physiological and pathological processes, with diverse roles in the brain ranging from neuronal development, homeostasis, and synaptic transmission to the harmful propagation of misfolded proteins in neurodegenerative diseases (Delpech et al., 2019).

EVs largely depend on their surface protein composition to interact with target cells and to deliver their signals and/or functional cargo (Buzás et al., 2018; Jahnke & Staufer, 2024). However, it remains unclear how—and importantly, when—target cell attachment and EV‐mediated signalling are modulated. With proteases as an integral part of their surface, the fate and function of EVs are not necessarily predetermined at the time of formation but may rather change dynamically during their journey through the extracellular space, shaped in part by proteolytic cleavage events. It is thus conceivable that cleavage of EV surface proteins directly impacts the mode, specificity, and speed of EV‐cell interactions, and that the proteolytic release of bioactive fragments from the EV surface affects recipient cells independent of direct EV‐to‐cell binding. In turn, this could likewise contribute to the spread of neurotoxic fragments or harmful protein assemblies in a disease context or create decoy fragments limiting therapeutic antibody efficacy (Hansen et al., 2016; Tosetti et al., 2018). Moreover, biomarker discovery relies on the detection of specific EV surface proteins (Buzás et al., 2018), which may be affected by continued protease activity on EVs.

Despite several studies having reported proteolytic activity on EVs and reviews pointing out its mechanistic and translational relevance (e.g., Quesnel et al., 2022; Sanderson et al., 2017; Shimoda, 2019; Shimoda and Khokha, 2013, 2017), the impact on EV biology and intercellular communication, as well as functional consequences, are largely unknown. ADAM10 is one of the most frequently detected sheddases associated with EVs and is considered a conserved and specific marker of small EVs (sEVs), which are generally < 200 nm in diameter, isolated by high‐speed centrifugation, and composed of small ectosomes derived from plasma membrane budding as well as of exosomes originating from the endocytic pathway (Crescitelli et al., 2020; Keerthikumar et al., 2015; Kowal et al., 2016). Specifically, ADAM10 is highly enriched on sEVs with low buoyant density, which are considered to represent exosomes based on predominantly containing proteins of endosomal origin (Crescitelli et al., 2020; Keerthikumar et al., 2015; Kowal et al., 2016). Moreover, ADAM10 on sEVs is mainly present in its mature form (i.e., lacking the autoinhibitory prodomain) and was shown to be functionally active in the context of immune responses and cancer, with identified substrates including L1CAM and CD44 (Stoeck et al., 2006), CD23 (Mathews et al., 2010), and CD30 (Hansen et al., 2016). Further, cleavage fragments of the amyloid precursor protein (APP) frequently detected in the EV membrane suggest continued ADAM10 activity on brain‐derived EVs (Lauritzen et al., 2019; Pérez‐González et al., 2020; Sharples et al., 2008). However, specific insights into the link between ADAM10 and sEVs in the brain, where both play central roles in development, plasticity, and disease, are lacking to date.

In this study, we investigated the proteolytic activity of ADAM10 on sEVs (sEV‐ADAM10) released by brain cell cultures and neuronal cell lines, hypothesising that its continued activity dynamically remodels the surface of EVs and modulates their composition, signalling potential, targeting, and adhesion properties in the brain.

## 2 Materials and Methods

### 2.1 Dissociated Rat Cortical Cultures

All animal experiments were performed with approval by the local authorities of the city‐state of Hamburg (*Behörde für Gesundheit und Verbraucherschutz, Fachbereich Veterinärwesen*) and the animal care committee of the University Medical Center Hamburg‐Eppendorf (UKE), Hamburg, Germany. Wistar Unilever HsdCpb:WU (Envigo) rats were obtained from the animal facility of the UKE Hamburg. Primary rat cortical cell cultures were prepared as previously described with slight modifications (Almansa et al., 2022; Laulagnier et al., 2017). Pregnant rats were sacrificed, and cortices were dissected from 18‐day‐old embryos (E18) and placed in cold Hibernate‐E medium supplemented with B27 and 0.5 mM glutamine. The cortices were disrupted by pipetting up and down once, washed with cold Hanks' Balanced Salt Solution (HBSS), treated with 0.25% trypsin‐EDTA for 20 min at 37°C, washed with warm HBSS, treated with 1 mg/mL DNase I, and physically dissociated by pipetting through a 20G and 26G cannula and a 100 µm cell strainer. The obtained cortical cells were seeded in plating medium (Dulbecco's Modified Eagle Medium (DMEM) supplemented with 10% fetal bovine serum (FBS) and 1% penicillin‐streptomycin) on 150 mm culture dishes for sEV isolation experiments or on glass coverslips in 6‐well plates for immunocytochemistry, both coated with poly‐L‐lysine (PLL). After 1 h, the plating medium was replaced with neuronal medium consisting of BrainPhys medium (StemCell, catalogue number (Cat#) 05790) supplemented with SM1 (StemCell, Cat# 05711) and 0.5 mM glutamine. Primary cultures were grown in an incubator at 37°C, 5% CO_2_, and 95% humidity.

### 2.2 Immunocytochemistry

Cortical cells were prepared as described above and seeded on PLL‐coated glass coverslips in 6‐well plates at 300,000 cells/well. At day in vitro (DIV) 13, cells were fixed with 4% paraformaldehyde and 4% sucrose in phosphate‐buffered saline (PBS) for 10 min, washed extensively with PBS, and permeabilised with 0.2% Triton X‐100 in PBS for 10 min at room temperature (RT). Cells were then washed with PBS, blocked for 1 h at RT with blocking buffer (PBS, 10% horse serum, 0.1% Triton X‐100), and incubated overnight at 4°C with primary antibodies (anti‐MAP2, 1:500, Sigma, Cat# M4403; anti‐GFAP, 1:500, Synaptic Systems, Cat# 173 002) in blocking buffer. Cells were washed three times with PBS, incubated with the corresponding fluorophore‐coupled secondary antibody (anti‐rabbit Alexa Fluor 488, 1:500, Invitrogen, Cat# A‐11034; anti‐mouse Alexa Fluor 568, Invitrogen, Cat# A‐11031) in blocking buffer for 1 h at RT in the dark, and washed again three times with PBS. The final PBS wash contained 1 µg/mL DAPI for 10 min to stain cell nuclei. Coverslips were mounted on microscope slides with Mowiol (Carl Roth, Cat# 0713.1) and left to dry. Fluorescence images were acquired by widefield microscopy with LED illumination on a Zeiss Axio Imager M2 using a 10× objective and standard fluorescent filters, and prepared using Fiji/ImageJ (Schindelin et al., 2012).

### 2.3 Isolation of sEVs From Primary Rat Cortical Cultures

sEVs were isolated from primary rat cortical cultures by differential ultracentrifugation as previously described (Laulagnier et al., 2017), with modifications. For sEV characterisation and sEV‐ADAM10 activity assays, 2.4 × 10^7^ seeded cortical cells were used from four 150 mm culture dishes (3.9 × 10^4^ cells/cm^2^). Cells were cultured for 13–15 days in 35 mL/dish of serum‐free neuronal medium (BrainPhys supplemented with SM1 and 0.5 mM glutamine). 5 mL of fresh neuronal medium were added every five days. The DIV13‐15 conditioned cell supernatant was collected, immediately put on ice, and supplemented with a cocktail of protease inhibitors (PIs) containing EDTA (Roche, Cat# 04693116001) for sEV characterisation or without PIs for sEV‐ADAM10 activity assays and N‐terminal proteomics. The supernatant was cleared of cell debris and large EVs by serial centrifugation at 2,000 × *g* for 10 min at 4°C, and then centrifuged again for 30 min at 4°C and 18,500 × *g*. Finally, the supernatant was filtered through a 0.22 µm filter into UltraClear ultracentrifuge tubes (Beckman‐Coulter, Cat# 344060) and sEVs were pelleted in an Optima L‐80 XP ultracentrifuge (Beckman‐Coulter) at 100,000 × *g* for 90 min at 4°C using an SW 40 Ti swinging‐bucket rotor (Beckman‐Coulter). The sEV pellet was resuspended in sEV collection buffer by 100× up‐and‐down pipetting using low‐binding filter tips. The sEV collection buffer comprised either PBS containing freshly added PIs for sEV characterisation, RIPA buffer (50 mM Tris‐HCl pH 8, 150 mM NaCl, 0.1% SDS, 0.5% Triton X‐100, 0.5% Na‐deoxycholate) with PIs for deglycosylation assays, or 20 mM HEPES for sEV‐ADAM10 activity experiments and N‐terminal proteomics. sEV isolates were either processed immediately for activity studies or stored at −80°C.

For N‐terminal proteomics experiments, 1.3 × 10^7^ seeded cells were used from two 150 mm culture dishes (4.3 × 10^4^ cells/cm^2^). For biological replicates, cortices of different embryos from the same or different litters were used. Per sEV preparation/biological replicate, 80 mL of cell supernatant were collected and processed as described above.

### 2.4 Preparation of Cell Lysates and Crude Membrane Fractions (CMFs)

Cell lysates from primary rat cortical cultures were prepared by washing cells in cold PBS, scraping and harvesting in cell lysis buffer (PBS, 1% Triton X‐100) with PIs or in RIPA buffer with PIs, incubating on ice for 20 min, centrifuging at 12,000 × *g* for 10 min at 4°C, and collecting the supernatant for storage at −80°C. For immunoblotting, cell lysates were diluted in self‐made 4× SDS sample loading buffer (250 mM Tris‐HCl, pH 6.8, 8% (w/v) SDS, 40% (v/v) glycerol, 5% (v/v) β‐mercaptoethanol, 0.004% bromophenol blue, pH 6.8), boiled at 95°C for 5 min, and stored at −20°C. CMFs from cultured primary rat cortical cells used for proteomic analysis were prepared by washing the cells in cold PBS, scraping and harvesting in CMF collection buffer (20 mM HEPES supplemented with PIs), and homogenisation by pipetting. Following centrifugation for 10 min at 1,000 × *g* at 4°C, the supernatant was collected and centrifuged again for 20 min at 12,000 × *g* at 4°C. Finally, the CMF‐containing pellet was resuspended in CMF collection buffer and stored at −80°C.

### 2.5 Nanoparticle Tracking Analysis (NTA)

Size distribution and concentration of isolated sEVs were measured based on their Brownian motion by NTA using a NanoSight LM14C instrument (Malvern Panalytical), equipped with an sCMOS‐type camera (model C11440‐50B/A11893‐02, Hamamatsu Photonics K.K.). sEV samples were diluted 1:100–1:500 in 0.6–1 mL of filtered PBS to yield a particle/frame count of 40–110 (Shearn et al., 2020). All sEV samples for functional experiments were run on the same day as collection, avoiding freeze‐thaw cycles. For comparing sEV particle changes after 24 h of incubation at 37°C, samples were collected from the top phase of the incubating sEV solution without prior mixing, subsequently diluted and immediately run on the NanoSight. Ten videos of 10 s length were recorded per sample at 25°C in light scatter mode with a camera level of 13 and a detection threshold of 8, and the videos were analysed using the batch processing function of the inbuilt NTA software (NTA 3.4 Build 3.4.003). For every different sEV resuspension buffer, sEV‐free buffer blanks were run to confirm the absence of particles. The collected data were prepared using GraphPad Prism 8 (Dotmatics).

### 2.6 SDS‐PAGE and Immunoblotting

For protein detection by immunoblotting, samples were diluted in self‐made 4× SDS sample loading buffer, boiled at 95°C for 5 min, and run by SDS‐PAGE on 4%–20% self‐made SDS polyacrylamide gels in SDS running buffer (192 mM glycine, 0.1% (w/v) SDS, 25 mM Tris‐base, pH 8.2). PageRuler (Invitrogen, Cat# 26617) was used as a pre‐stained protein ladder. All sEV samples across and within groups were loaded based on equal volume. Proteins were transferred onto activated polyvinylidene fluoride (PVDF) membranes (BioRad, Cat# 1620177) in blotting buffer (192 mM glycine, 0.1% (w/v) SDS, 15% (v/v) methanol, 25 mM Tris‐base, pH 8.3) using a wet blot system at a constant voltage of 110 V for 1 h. Membranes were blocked with 5% milk powder in Tris‐buffered saline containing Tween‐20 (20 mM Tris pH 7.4, 150 mM NaCl, 0.1% Tween‐20) (TBS‐T) for 1 h at RT and incubated overnight at 4°C with primary antibodies against ADAM10 (polyclonal, C‐terminal, 1:1000, Abcam, Cat# ab1997; clone EPR5622, C‐terminal, 1:1000, Abcam, Cat# ab124695), ALIX (clone E6P9B, 1:1000, Cell Signalling, Cat# 92880), flotillin‐1 (clone 18/Flotillin‐1, 1:1000, BD Biosciences, Cat# 610820), GM130 (clone 35/GM130 (RUO), 1:500, BD Biosciences, Cat# 610823), LC3B (polyclonal, 1:1000, Abcam, Cat# ab48394), APP and APP‐CTFs (clone Y188, 1:1000, Abcam, Cat# ab32136), LRP1 (clone 11H4, 1:1000, BioLegend, Cat# 858701), or NCAM1 (polyclonal, 1:1000, R&D, Cat# AF2408). Corresponding secondary antibodies conjugated with horseradish peroxidase (HRP) (anti‐mouse and anti‐rabbit, 1:10,000, Jackson ImmunoResearch; anti‐goat, 1:25,000, Sigma, Cat# A5420) were applied for 1 h at RT in TBS‐T containing 5% milk powder. Membranes were developed using an enhanced chemiluminescence solution made in‐house (Haan and Behrmann, 2007) on a ChemoStar imager (Intas).

### 2.7 Protein Quantification

sEV samples in 20 mM HEPES buffer were thawed from −20°C and lysed by adding 1% Triton X‐100 for 30 min at RT, and the protein amount in sEV and CMF samples was quantified prior to N‐terminal proteomics using a micro bicinchoninic acid (BCA) protein assay (Thermo Scientific) according to the manufacturer's instructions. The absorbance was measured at 562 nm using a TECAN Infinite 200 PRO reader (Tecan Group Ltd.).

### 2.8 Deglycosylation of Samples (PNGase F Digestion)

Following manufacturer's instructions, *N*‐linked glycans were removed from sEV and cell lysate samples using the PNGase F kit (New England Biolabs, Cat# P0704). Cell lysates and sEVs in RIPA buffer with PIs were thawed from −80°C, and 1.5 µg of protein was denatured with GlycoBuffer 1 for 10 min at 100°C and chilled on ice for 5 min before incubating with GlycoBuffer 2, 10% NP‐40, and peptide:*N*‐glycosidase F (PNGase F) at 37°C for 2 h. Samples were then diluted in 4× SDS sample loading buffer, boiled at 95°C for 5 min, and frozen at −20°C. For immunoblot analyses, 0.7 µg per PNGase F‐treated or untreated sample was loaded.

### 2.9 Transmission Electron Microscopy (TEM)

To visualise overall sEV morphology, negative staining in combination with TEM was performed as described in Théry et al. (2006). Briefly, sEVs were isolated as above, and the sEV pellet was resuspended and fixed in 2% paraformaldehyde. Carbon‐coated copper grids (EMS, Cat# CF200‐CU) were glow‐discharged at 25 mA for 60 s to hydrophilise the surface (Quorum Technologies). Thereafter, 5 µL of resuspended sEVs were adsorbed onto the grids for 20 min. After washing with PBS, samples were postfixed with 1% glutaraldehyde in PBS and incubated in ice‐cold methylcellulose‐uranyl acetate solution for 30 min. Grids were looped out, air‐dried, and analysed by electron microscopy. Images were acquired at 200 kV with a JEM‐2100Plus transmission electron microscope (Jeol) equipped with an XAROSA CMOS camera (Emsis).

### 2.10 Fluorogenic Peptide‑Based sEV‐ADAM10 Activity Assay

The proteolytic activity of sEV‐ADAM10 was analysed using the ADAM10‐selective fluorogenic substrate PEPDAB063 (Acetyl‐dArg(3)‐dGlu(3)‐hexaminoyl‐K(Dabcyl)‐PRYEAYKMGK(5FAM)‐NH2) from BioZyme, Inc. (Caescu et al., 2009). sEVs were isolated from primary rat cortical cells as described above but without the use of PIs in the cell supernatant. Immediately after sEV isolation, sEVs were resuspended in cold 20 mM HEPES buffer (pH 7.0–7.6), split into equal parts by volume, and pre‐incubated for 30 min with either the ADAM10‐selective hydroxamate‐based inhibitor GI254023X (GI) (Ludwig et al., 2005) (Selleck Chemicals) in DMSO (5 µM final concentration) or a control containing the same concentration of DMSO in 20 mM HEPES. sEV samples were transferred to black, flat‐bottom, non‐binding 96‐well plates, and fluorogenic PEPDAB063 was added simultaneously to all wells (10 µM final concentration). Activity assays were run at 37°C on a TECAN Spark 10 M (Tecan Group Ltd.), and fluorescence was measured every 10 min for 3 h at excitation/emission wavelengths of 485/535 nm. A 30‐minute equilibration period was allowed before measurements were considered. For end‐point determination, plates with samples were kept at 37°C, and fluorescence was measured again after 24 h. Three independent experiments were performed. Each assay included sEV‐free background wells containing only buffer, substrate, and GI or DMSO control.

### 2.11 Proteomics and N‐Terminal Proteomics

#### 2.11.1 Sample Preparation for Mass Spectrometry

Four biological replicates of cortical culture‐derived sEVs and CMFs were prepared as described above. To ensure identical vesicle populations between conditions, the same sEV preparation was split equally into three groups. sEVs were either used freshly prepared (0 h) or incubated for 24 h at 37°C in 20 mM HEPES with 5 µM GI or DMSO control. Samples were diluted with SDS sample loading buffer and boiled for 5 min at 95°C. CMFs from each of the biological replicates were pooled to serve as a reference to the sEV samples and analysed in parallel. Each sEV sample contained roughly 10 µg of protein material, while the pooled CMF sample contained 125 µg of total protein. Samples were reduced and alkylated with tris(2‐carboxyethyl)phosphine (5 mM) and iodoacetamide (15 mM), respectively. The reaction was quenched with dithiothreitol (15 mM) before precipitation on hydrophilic and hydrophobic Sera‐Mag SpeedBeads (carboxylate‐modified magnetic beads, GE Healthcare) using a 6‐fold volume of ethanol. The beads were washed twice with 90% ethanol. Primary amines (protein N‐terminus and lysine residues) were reductively dimethylated with a labelling solution consisting of 30 mM formaldehyde and 15 mM sodium cyanoborohydride in HEPES buffer (200 mM, pH 7) containing 4 M guanidine hydrochloride. Samples were left to react for 3 h at 37°C before another aliquot of labelling reagents was added and left to incubate overnight at 37°C. The labelling reaction was quenched by adding 0.9 M Tris‐HCl for 3 h. Samples were then precipitated on fresh SpeedBeads as described above and then digested overnight at 37°C with 200 ng of trypsin (1:50, enzyme:protein) in 100 mM triethylammonium bicarbonate buffer. Twenty percent (approximately 2 µg) of the sample was set aside for ‘PRE‐HYTANE’ while the rest of the sample was taken and the ‘neo’ N‐termini (generated as a result of trypsin digestion) were depleted using HYTANE (*HYdrophobic Tagging‐Assisted N‐termini Enrichment*). Samples were redissolved in HEPES buffer (pH 7) and 500 µg hexadecanal (50:1, w/w ratio of hexadecanal to peptide) was added along with 30 mM sodium cyanoborohydride in the presence of isopropanol (overnight at 42°C). A new aliquot of sodium cyanoborohydride was added and samples were dried for 2 h via vacuum evaporation at 60°C. Samples were acidified by diluting to 600 µL with 0.1% trifluoroacetic acid (TFA), and any unprecipitated hexadecanal was captured on a C‐18 column. Peptides were eluted off the column with 80% acetonitrile (ACN) in the presence of 0.1% TFA. Samples were dried (vacuum evaporation) and stored at −20°C until analysis.

#### 2.11.2 Liquid Chromatography‐Mass Spectrometry (LC‐MS) Measurements

Samples were injected in duplicate on a Dionex Ultimate 3000 nano‐UHPLC coupled to a Q Exactive Plus mass spectrometer (Thermo Scientific). Samples were washed on a trap column (Acclaim Pepmap 100 C‐18, 5 mm × 300 µm, 5 µm, 100 Å, for the Dionex Ultimate 3000 nano‐UPLC or a 20 mm × 75 µm, 100 Å, for the EASY‐nLC) with 3% ACN/0.1% TFA before peptide separation using an Acclaim PepMap 100 C18 analytical column (50 cm × 75 µm, 2 µm, 100 Å, Dionex). A flow rate of 300 nL/min using eluent A (0.05% formic acid) and eluent B (80% ACN/0.04% formic acid) was used for gradient separation (120‐minute gradient, 5%–40% B). Spray voltage applied on a metal‐coated PicoTip emitter (10 µm tip size, New Objective) was 1.7‐1.9 kV, with a source temperature of 250°C. Full scan MS spectra were acquired between 375 and 1,400 m/z at a resolution of 70,000 at m/z 200, and the 10 most intense precursors with charge states greater than 2+ were selected for fragmentation using an isolation window of 1.4 m/z and higher energy collision dissociation (HCD) normalised collision energies of 27 at a resolution of 17,500. Lock mass (445.120025) and dynamic exclusion (30 s) were enabled.

#### 2.11.3 Database Search and Statistics

The MS raw files were processed by Proteome Discoverer 2.2 and MS/MS spectra were searched using the Sequest HT algorithm against a database containing common contaminants and the complete *Rattus norvegicus* database (reviewed and unreviewed, 29,935 entries). The enzyme specificity was set to semi‐ArgC, and two missed cleavages were allowed. An MS1 tolerance of 10 ppm and a MS2 tolerance of 0.02 Da was implemented. Oxidation (15.995 Da) of methionine residues, acetylation (42.011 Da), methylation (14.016 Da) and dimethylation (28.031 Da) on the peptide N‐terminus was set as a variable modification. In comparison, carbamidomethylation (57.02146 Da) on cysteine residues and dimethylation on lysine residues was set as a static modification. Minimal peptide length was set to 7 amino acids and the peptide false discovery rate (FDR) was set to 1%. Raw abundances from Proteome Discoverer were exported, median normalised and log_2_‐transformed in Excel. Student's *t*‐tests were performed in Excel.

#### 2.11.4 Bioinformatic Analysis

The data were filtered to only include proteins that *(i)* were found in at least 2 of 4 biological replicates in every condition, *(ii)* have a peptide number of 2 or higher, and *(iii)* are specific to *Rattus norvegicus*. Protein sets were searched for common markers of brain cell types and known ADAM10 substrates, with Gene Ontology (GO) annotations stemming from the DAVID database (Huang et al., 2009; Sherman et al., 2022). Venn diagrams were created with InteractiVenn (Heberle et al., 2015). Hierarchical cluster analysis comparing the N‐terminal proteome of sEVs was performed with averaged replicate values of significantly downregulated proteins following 24 h sEV‐ADAM10 inhibition (compared to untreated controls) and visualised as heatmaps using Partek Genomics Suite (Partek). Volcano plots were created with GraphPad Prism 9 (Dotmatics). Topology annotations were extracted from supplementary databases from Kuhn et al. (2016) and Cvjetkovic et al. (2016). Protein class annotations were taken from UniProt (https://www.uniprot.org/) and the Panther Classification System (Thomas et al., 2022). Identified peptide sequences were compared to mouse‐ and human‐derived peptides reported in Ameen et al. (2023) and Tsumagari et al. (2021), respectively.

### 2.12 Statistical Analysis

Statistical analysis was performed using GraphPad Prism 8 and 9 (Dotmatics), with detailed information for each experiment indicated in the respective figure legends. For the sEV fluorescence assay, background wells were subtracted and the means of three independent experiments were calculated. Unpaired two‐tailed Student's *t*‐test was used to compare between time points in each group. NTA data for sEV stability over time were analysed by ordinary one‐way ANOVA (Tukey's multiple comparisons test, unpaired). Statistical significance was considered from *p* values < 0.05.

## 3 Results

### 3.1 Isolation and Characterisation of sEVs from Cultured Primary Rat Cortical Cells

**Figure 1:**
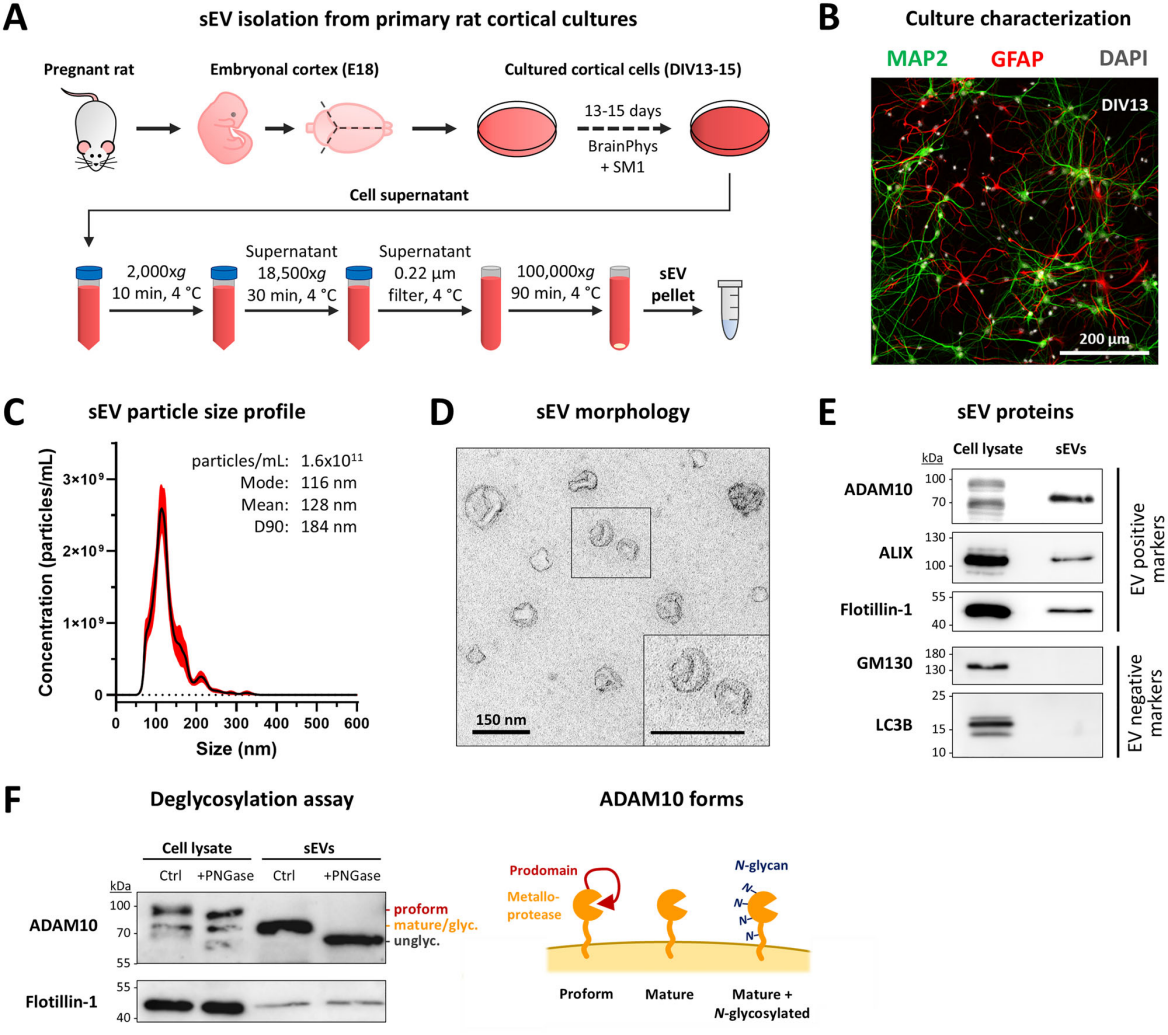
sEVs derived from cortical cultures carry mature, *N*‐glycosylated ADAM10. (A) Schematic representation of the sEV isolation protocol. sEVs were isolated from the cell supernatant of DIV13‐15 primary rat cortical cell cultures by serial centrifugation including a 0.22 µm‐filtration step. (B) Representative fluorescence microscopy image of mixed cortical cells showing a composition of MAP2‐positive neurons and GFAP‐positive astrocytes. DAPI was used to detect the nuclei of all cells. Scale bar: 200 µm. (C) Representative NTA plot showing the size distribution and concentration of the isolated sEVs. The red line indicates SEM. (D) Representative TEM image of isolated sEVs prepared with negative staining. Scale bar: 150 nm. (E) Representative immunoblots of cell lysates and isolated sEVs from DIV13 cortical cell cultures. ADAM10 (C‐terminal antibody, Abcam, Cat# ab1997), ALIX, and flotillin‐1 served as ‘positive’ EV markers, whereas GM130 and LC3B served as intracellular markers to assess the purity of sEV isolations. (F) Deglycosylation of cell lysate and sEVs. Lysed cells and sEVs were treated with PNGase F or vehicle control for 2 h and probed by immunoblot with an antibody against the C‐terminus of ADAM10. Detection of flotillin‐1 was used as loading control. The scheme shows different maturation forms of ADAM10, highlighting its four potential *N*‐glycosylation sites. Glyc. = *N*‐glycosylated; unglyc. = unglycosylated.

To study the role of ADAM10 in EV‐mediated brain cell communication, we established protocols for the isolation and characterisation of sEVs from primary rat cortical cell cultures (Figure 1A). Dissociated cells from cortices of rat embryos were grown for 13–15 days in synthetic, serum‐free BrainPhys medium. The mature cortical cultures were composed of a network of MAP2‐positive neurons, GFAP‐positive astrocytes, and other non‐neuronal cells (DAPI‐positive nuclei, but MAP2‐ and GFAP‐negative) (Figure 1B), in line with reports using a similar protocol (Almansa et al., 2022). We decided to maintain the mix of neuronal/glia cells to better reflect physiological complexity, retain natural communication of sEVs from different cell types, and avoid stress responses by selecting for certain isolated subtypes. sEVs were purified from conditioned cell supernatants by serial centrifugation and subsequently characterised. NTA showed average concentrations of > 10^11^ particles/mL and an expected size distribution between 50 and 220 nm (Figure 1C). TEM illustrated the vesicular morphology of the particles, as indicated by the cup‐shaped appearance characteristic for EVs analysed with this method (Raposo & Stoorvogel, 2013) (Figure 1D). Immunoblot analysis confirmed that the vesicles are extra‐ rather than intracellular vesicles, as they contained EV‐positive markers ADAM10, ALIX, and flotillin‐1, while lacking negative markers GM130 (a Golgi marker) and LC3B (an autophagosome marker) (Figure 1E). Notably, ADAM10 in sEV samples was identified to be primarily present in its mature (and often considered ‘active’) form (here: ∼70 kDa), rather than its autoinhibitory prodomain‐containing proform (here: ∼100 kDa) (Figure 1F). Moreover, we found sEV‐ADAM10 from brain cell cultures to be glycosylated with *N*‐glycans as evidenced by PNGase digestion (Figure 1F), in line with a previous report using a non‐neuronal cell line model (Escrevente et al., 2008). Since only the mature form is proteolytically active and *N*‐glycosylation protects from degradation (Escrevente et al., 2008), both features are expected prerequisites for ADAM10 to be enzymatically active on sEVs.

### 3.2 Brain Cell‐Derived sEV‐ADAM10 is Active towards a Synthetic Substrate

**Figure 2:**
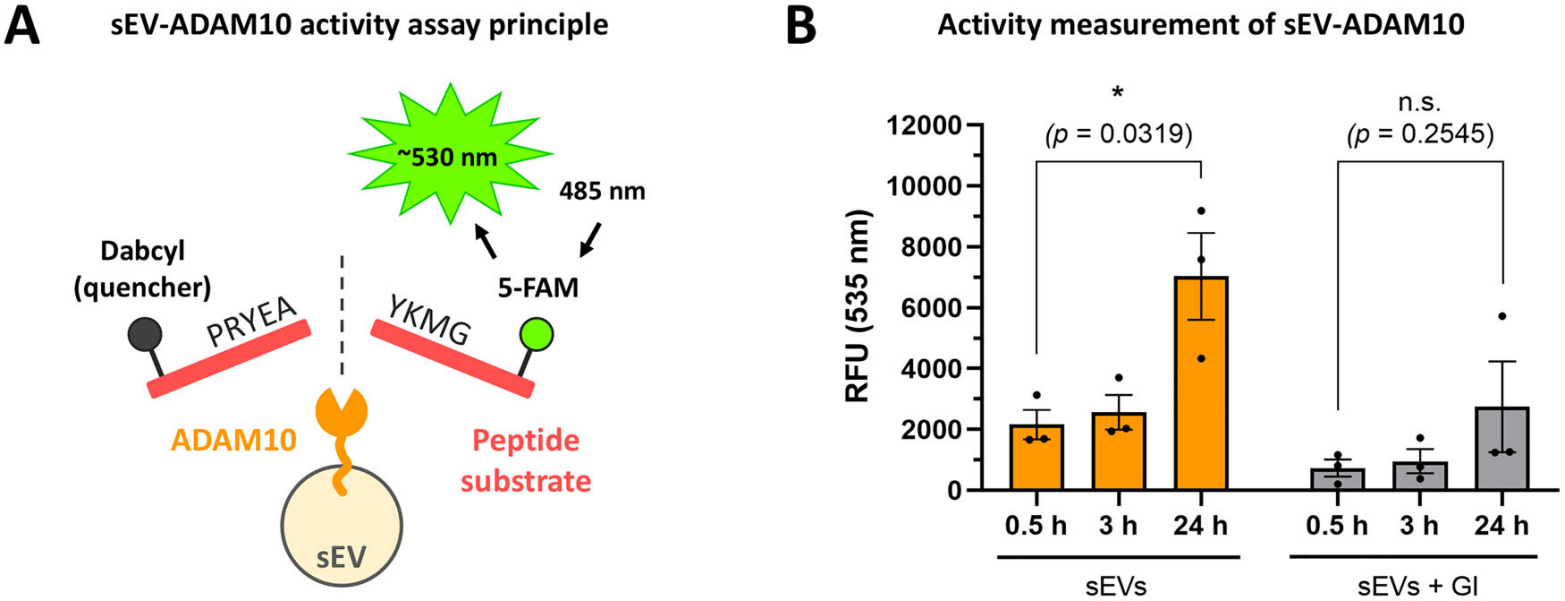
Brain cell‐derived sEV‐ADAM10 is active up to 24 h and cleaves an exogenous peptide substrate. Isolated cortical culture‐derived sEVs were incubated at 37°C with a fluorogenic ADAM10 substrate (‘PEPDAB063’, BioZyme, Inc.) in the presence or absence of ADAM10 inhibitor GI254023X (GI). (A) Schematic representation of the fluorogenic substrate assay, where ADAM10‐mediated cleavage of the peptide separates the quencher (Dabcyl) from the fluorophore (5‐FAM), resulting in increased fluorescence emission (~530 nm). (B) sEV‐ADAM10 activity measurement was performed at 0.5, 3, and 24 h, with or without GI. Fluorescence was measured at 535 nm, and the bar chart shows the mean relative fluorescence units (RFUs) at each time point, together with individual replicates and error bars representing the SEM. Two‐tailed Student's *t*‐test, *p < 0.05, n.s. = not significant, *n*  =  3.

As protease activity is the result of a finely tuned interplay of many different factors and the mere presence of mature ADAM10 on EVs does not per se guarantee its activity there (Aljohmani et al., 2022; Lambrecht et al., 2018), we performed cell‐free activity assays with the purified sEVs using a synthetic and fluorogenic ADAM10 substrate (PEPDAB063, BioZyme, Inc.) in the presence or absence of the selective ADAM10 inhibitor GI254023X (GI) (Figure 2A). sEVs were isolated from cultured cortical cells as before but in the absence of a PI cocktail in the cell supernatants. Incubation of sEVs with the ADAM10 substrate led to a slow but steady increase in fluorescence signal in untreated sEV samples compared to GI‐treated sEVs over 3 h (Figure 2B). When measuring the fluorescence after 24 h, the signal in untreated but not in GI‐treated sEVs had increased significantly (*p*  =  0.0319). The combination of employing a selective ADAM10 substrate and inhibitor suggests that the majority of signal from isolated cortical culture‐derived sEVs was generated by sEV‐ADAM10 activity, which appeared to be consistent over a relatively long period of time.

### 3.3 Known ADAM10 Substrates Present in the sEV Proteome Correlate With Major Biological Processes Associated With sEVs

**Figure 3:**
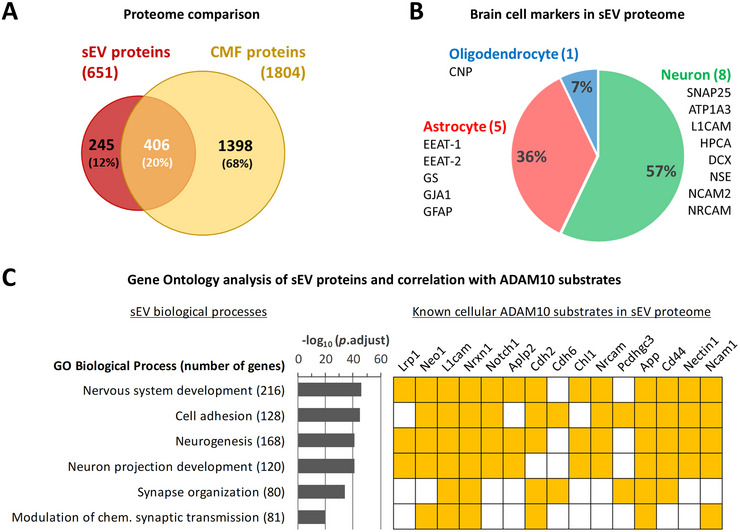
The sEV proteome contains known cellular ADAM10 substrates correlating with sEV‐mediated biological processes. Mass spectrometric analysis was performed with isolated sEVs derived from rat cortical cultures (DIV13‐15) (*n* = 4). (A) Venn diagram comparing the number of proteins detected in the sEV samples with the number of proteins from the CMFs of the donor cells. (B) Pie chart displaying the presence and proportion of identified brain cell type‐associated markers in sEV samples. (C) GO analysis of the sEV proteome for biological processes using DAVID. Selected GO terms with the most significant adjusted *p* values (Benjamini correction) are plotted against their −log10 (p.adjust). Associated proteins include known cellular ADAM10 substrates identified in the sEV samples. Orange boxes indicate a connection between the GO term and the corresponding protein.

Since ADAM10 demonstrated general activity in isolated sEV samples, we next asked whether and which potential ADAM10 substrates are present on sEVs that could be modulated by sEV‐ADAM10 activity. To screen the sEV proteome, we performed mass spectrometric analysis of sEVs isolated from cortical cultures. CMFs derived from the sEV‐secreting cells were run as a reference, yielding a total of 1,804 proteins (Figure 3A, Table S1). For sEV samples, a total of 651 proteins was identified. The sEV proteome contained several brain cell type markers relating to neurons, astrocytes, and oligodendrocytes, but not microglia (Figure 3B), thus fittingly reflecting the mixed composition of the sEV‐secreting cortical cell cultures (Figure 1B).

Analysis of GO biological process terms for sEV proteins revealed that proteins involved in neuronal developmental processes, cell adhesion, and synaptic functions were among the most significantly overrepresented (Figure 3C, Table S2). Surprisingly, when comparing with previously reported cellular ADAM10 substrates (Table 1), we found a substantial overlap with many of the biological processes connected to sEVs (Figure 3C). These ADAM10 substrates included L1CAM, APP, N‐cadherin (CDH2), and NCAM1, with prominent roles in cell adhesion, synaptic transmission, and nervous system development, including neurogenesis. Further, cell adhesion molecules with functions in synapse organisation such as protocadherin gamma C3 (PCDHGC3), neurexin‐1 (NRXN1), CDH6, and CD44 were detected in the sEVs. Other identified ADAM10 substrates included LRP1—the most abundant protein in the dataset—, NOTCH1, APLP2, neogenin‐1 (NEO1), and nectin1, with functions in neuron projection development and other developmental processes (Figure 3C, Table S2).

**Table 1:** Selection of known cellular ADAM10 substrates identified in sEV samples from primary rat cortical cell cultures.

Protein name	Gene symbol	Accession	Unique peptides	PSMs	Topology	ADAM10 substrate reference
Prolow‐density lipoprotein receptor‐related protein 1	*Lrp1*	G3V928	40	489	Type I	Liu et al., 2009
Neogenin	*Neo1*	P97603	16	266	Type I	Kuhn et al., 2016
Neural cell adhesion molecule L1	*L1cam*	A0A0G2KA95	10	206	Type I	Mechtersheimer et al., 2001
Neurexin‐1	*Nrxn1*	Q63372	9	87	Type I	Trotter et al., 2019
Neurogenic locus notch homolog protein 1	*Notch1*	Q07008	7	56	Type I	Hartmann et al., 2002
Amyloid precursor‐like protein 2	*Aplp2*	P15943	6	86	Type I	Endres et al., 2005
Cadherin‐2	*Cdh2*	G3V803	5	50	Type I	Reiss et al., 2005
Cadherin‐6	*Cdh6*	F1LQP8	4	51	Type I	Kuhn et al., 2016
Neural cell adhesion molecule L1‐like protein	*Chl1*	M0RC17	4	46	Type I	Kuhn et al., 2016
Neuronal cell adhesion molecule	*Nrcam*	A0A0G2K3Q5	4	28	Type I	Kuhn et al., 2016
Protocadherin gamma C3	*Pcdhgc3*	A0A0G2K9D7	4	111	Type I	Reiss et al., 2006
Amyloid precursor protein	*App*	P08592	3	13	Type I	Jorissen et al., 2010; Kuhn et al., 2010
CD44 antigen	*Cd44*	F1LSA1	2	36	Type I	Nagano et al., 2004
Nectin cell adhesion molecule 1	*Nectin1*	F1LNP8	2	17	Type I	Kim et al., 2010
Neural cell adhesion molecule 1	*Ncam1*	P13596	1	165	GPI/Type I	Brennaman et al., 2014

To relate our proteomics data to published reports, we compared our protein set with two available proteome lists: one for EVs isolated by ultracentrifugation from primary rat hippocampal neuron cultures (Vilcaes et al., 2021) and one for exosomes isolated from human frontal cortex (Vella et al., 2017) (Figure S1, Table S3). We found 243 proteins to be present in all three datasets and—besides the EV markers flotillin‐1, ALIX, syntenin‐1, and ADAM10—the list contained many of the same ADAM10 substrates we could previously associate with cell adhesion, modulation of chemical synaptic transmission, neuron projection development, and synapse organisation (notably APP, L1CAM, CDH2, NRXN1, and CD44). This correlation suggests that the identified ADAM10 substrates and the associated biological processes are conserved in EVs across different samples and literature reports, with translational relevance to human brain tissue.

Lastly, we confirmed the presence of the ADAM10 substrates APP, LRP1, and NCAM1 in our sEV samples by immunoblotting (Figure S2). Moreover, we detected C‐terminal fragments for APP, indicative of proteolytic cleavage activity either before or after sEV secretion.

Taken together, it appears that major biological processes associated with sEVs, such as cell adhesion, synaptic functions, and neuronal development, correlate with the functional spectrum of ADAM10 substrates. This relationship links ADAM10 to EV function, with continuous sEV‐ADAM10 activity towards these proteins possibly affecting EV‐cell interaction properties and signalling actions of sEVs.

### 3.4 sEV‐ADAM10 Activity Changes sEV Surface Proteins Involved in Neuronal Development, Signalling, and Cell Adhesion

To scrutinise sEV‐ADAM10 activity towards endogenous sEV proteins and possibly identify new sEV‐ADAM10 substrate candidates, we examined the generation of N‐terminal fragments from sEVs in cell‐free assays over time. We selected a 24 h endpoint to increase the observable effects and assessed whether the sEVs remain stable over this period using NTA and immunoblotting. Comparison of freshly prepared sEVs (0 h) with sEVs incubated for 24 h at 37°C in the absence or presence of GI indicated the presence of intact sEVs by showing no significant changes in particle size and number (Figure S3A and B) and by yielding similar band intensities for ADAM10 and EV markers ALIX and flotillin‐1 (Figure S3C).

**Figure 4:**
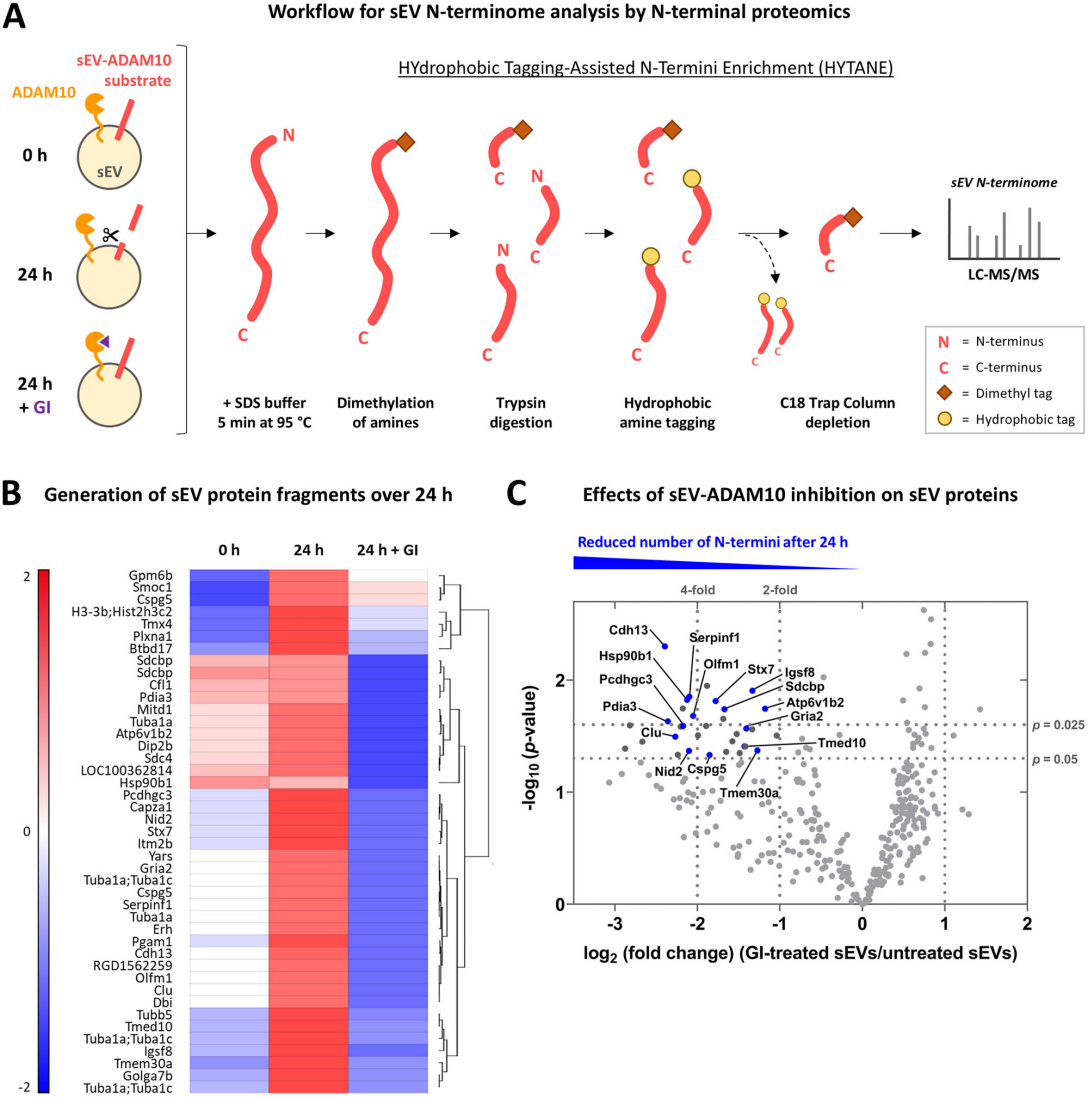
Inhibition of sEV‐ADAM10 reduces the generation of N‐terminal protein fragments. (A) Schematic representation of the workflow for N‐terminal proteomics. sEVs were isolated from the supernatant of DIV13–15 primary cortical cells and either used immediately (0 h) or incubated at 37°C in 20 mM HEPES buffer for 24 h in presence or absence of the ADAM10 inhibitor GI. The samples were transferred to SDS sample loading buffer and subjected to N‐terminal proteomics for the detection of N‐terminal fragments generated over time (*n* = 4 per group). (B) Heatmap comparing significantly altered protein fragments between 0 h, 24 h untreated, and 24 h treated with GI. The colour key represents the normalised abundance level. Red shows an increase of fragments, blue shows a reduction of fragments, white shows no change. Multiple entries of the same protein indicate detection of different fragments varying in sequence or modification. (C) Volcano plot of N‐terminal fragments in sEV samples treated with GI for 24 h compared to untreated samples after 24 h. Depicted is the −log_10_ (*p* value) against the log_2_ (fold change), with significance and fold change thresholds indicated. Selected candidates used for further analysis are highlighted in blue.

For detection of newly generated N‐terminal protein fragments from the sEVs, we combined mass spectrometry with a *hydrophobic tagging‐assisted N‐termini enrichment strategy* (‘HYTANE’) (L. Chen et al., 2016), allowing for analysis of the sEV N‐terminome (Figure 4A). After tagging of N‐termini and depletion of fragments generated by trypsin during sample preparation for LC‐MS, 323 N‐terminal peptides were identified in at least two of four biological replicates across all three groups. An increased number of N‐terminal fragments was detected for several sEV proteins after 24 h compared to 0 h, suggesting their release following proteolytic processing on sEVs (Figure 4B). In contrast, the same proteins did not show an increase in N‐terminal fragment generation in sEV samples treated with GI over 24 h, hence implying ADAM10‐mediated effects (Figure 4B). Comparing only the GI‐treated group with the untreated control group yielded a panel of 33 proteins with a reduced number of N‐terminal fragments after 24 h of ADAM10 inhibition (*p* < 0.05) (Figure 4C, Table S4). Among the significantly altered proteins were several cytosolic proteins such as tubulins TUBA1A and TUBB5, nuclear molecules DIP2B and ERH, and other intracellular proteins like CAPZA1, GOLGA7B, and YARS (Table S4). Although many of the tubulins and other cytoplasmic proteins are not uncommon in mass spectrometry data of EV samples (e.g., Vella et al., 2017, and Vilcaes et al., 2021), it was unexpected to find them changed in relation to sEV‐ADAM10 inhibition. As these proteins are considered part of the intraluminal cargo of EVs, and as sEVs remained intact over the incubation period (Figure S3), sEV surface‐bound ADAM10 would not have had spatial access to process them. Though it is possible that the cytosolic proteins have become part of the external EV protein corona during EV purification, we reasoned that they are not representing likely physiological substrates for ADAM10 and excluded them from further analysis.

**Figure 5:**
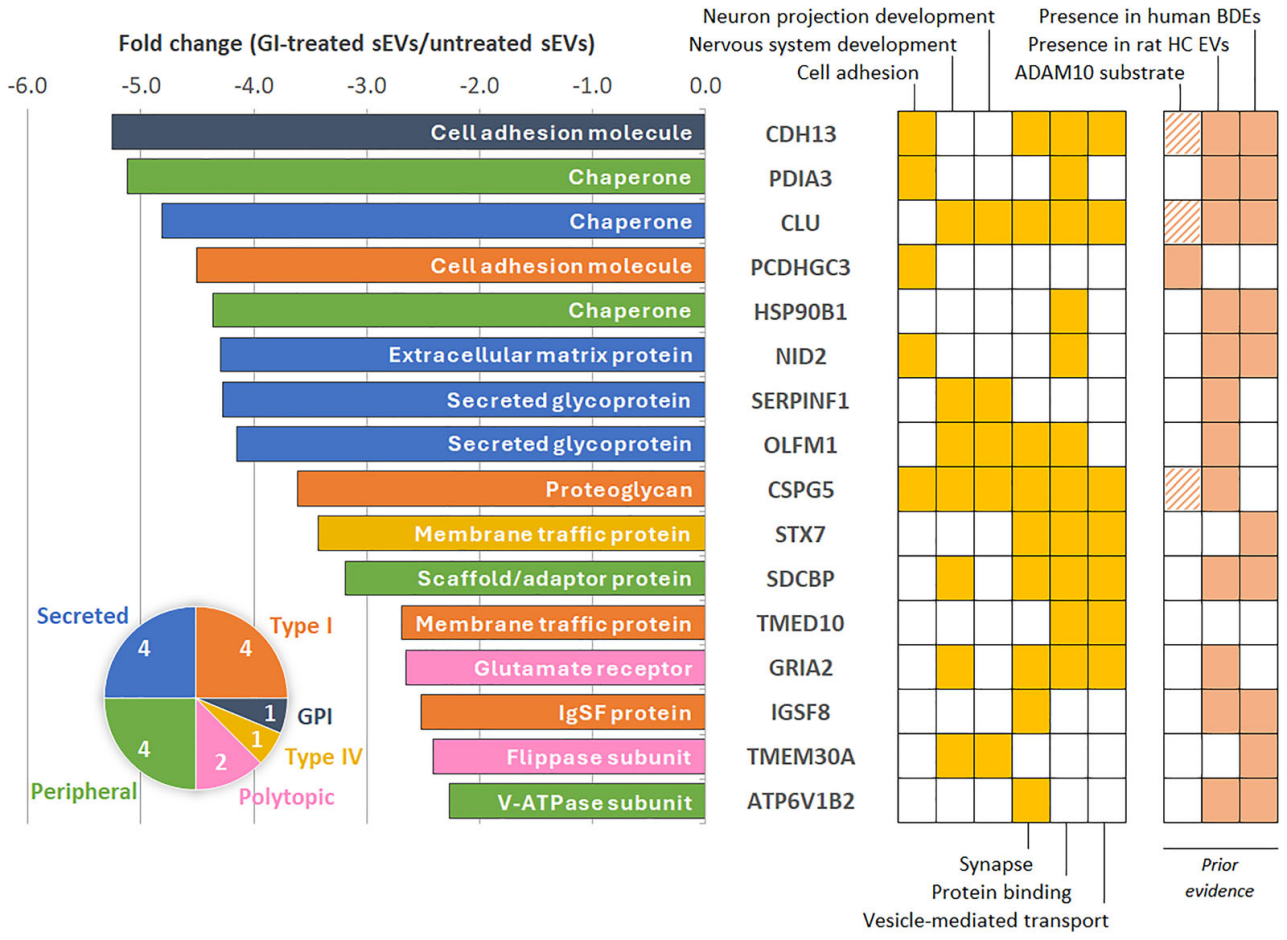
sEV‐ADAM10 inhibition affects sEV surface proteins involved in neuronal development, signalling, and cell adhesion. Membrane‐associated proteins with the most significantly reduced number of N‐terminal fragments after 24 h of sEV‐ADAM10 inhibition were bioinformatically analysed for topological and functional features (*n* = 4 per group). Bar chart: Proteins are shown against their fold change reduction of fragment number following treatment with the ADAM10 inhibitor GI compared to untreated. Each bar contains the main protein class (PANTHER/UniProt) of the respective protein. The bar colour corresponds to the topology of the protein as indicated in the pie chart. Pie chart: Analysis of the annotated cellular topology of the 16 proteins, showing the distribution of type I and type IV membrane, polytopic, peripheral, GPI‐anchored, and secreted proteins, with the number of proteins in each category indicated within the pie segments. Annotation matrix: Descriptive Gene Ontology analysis (DAVID) of selected biological processes, cellular components, and molecular functions, with orange boxes illustrating a connection between the protein and the corresponding GO term. Boxes with diagonal hatching indicate proteins previously identified as ADAM10 substrate candidates in a proteomic study. Light red boxes indicate previously confirmed cellular ADAM10 substrates or proteins present in proteomic datasets of rat hippocampal (HC) neuron EVs (Vilcaes et al., 2021) or human brain‐derived exosomes (BDEs) (Vella et al., 2017).

The remaining sixteen significantly altered membrane‐associated proteins affected by sEV‐ADAM10 activity (as judged by changes upon GI treatment) comprised four type I membrane proteins, four secreted proteins, four peripheral proteins, two polytopic proteins, one type IV membrane protein, and one glycosylphosphatidylinositol (GPI)‐anchored protein (Figure 5, left). Among the proteins that were more than 3‐fold decreased upon sEV‐ADAM10 inhibition, we found cell adhesion molecules, chaperones, and membrane trafficking‐associated proteins, with the most significantly altered protein being GPI‐anchored T‐cadherin/cadherin‐13 (CDH13) (Table 2). GO analysis of biological processes, cellular components, and molecular functions highlighted major links to neuronal function and localisation (Figure 5, right). More than half of the proteins were synapse‐associated, with several being part of the presynaptic membrane or glutamatergic or GABAergic synapses (Table S4). Clusterin (CLU), serpin F1/PEDF (SERPINF1), noelin/pancortin (OLFM1), neuroglycan C/chondroitin sulfate proteoglycan 5 (CSPG5), and TMEM30A are involved in neuronal differentiation, nervous system development, and neuron projection development. Likewise prominent were candidates with protein transport features pertaining to roles in vesicle‐mediated transport and fusion with the plasma membrane, such as the membrane trafficking‐associated proteins syntaxin‐7 (STX7), syntenin‐1 (SDCBP), and TMED10. Moreover, most identified candidates have protein‐binding properties, with five proteins being involved in cell‐cell adhesion or cell‐matrix adhesion, namely CDH13, PDIA3, PCDHGC3, nidogen‐2 (NID2), and CSPG5 (Figure 5, Table S4). PCDHGC3 itself was previously confirmed to be an ADAM10 substrate (Reiss et al., 2006) and three other proteins (CDH13, CLU, and CSPG5) were included among ADAM10 substrate candidates identified in a previous proteomics study (Kuhn et al., 2016). In addition, many of the proteins identified in the present study were found in available proteome lists of EVs from rat hippocampal neuron cultures and exosomes from human frontal cortex (Figure 5, Table S3).

**Table 2:** Significantly altered proteins after 24 h of sEV‐ADAM10 inhibition, and their detected peptides.

Protein name	Gene symbol	*p* value (GI/ctrl)	Fold change (GI/ctrl)	Identified amino acid sequence (N‐terminus to C‐terminus)^1^	Identified peptide	Expected N‐terminal start of the mature protein^2^
Cadherin‐13	*Cdh13*	0.00499	−5.26	[R].SIVVSPILIPENQR.[Q]	139–152 aa	139
Clusterin	*Clu*	0.03192	−4.82	[G].EQEFSDNELQELSTQGSR.[Y]	22–39 aa	22
Glutamate receptor	*Gria2*	0.02700	−2.65	[S].NSIQIGGLFPR.[G]	25–35 aa	25
Noelin	*Olfm1*	0.02082	−4.15	[P].TNPEESWQVYSSAQDSEGR.[C]	54–72 aa	17
Syntenin‐1	*Sdcbp*	0.01817	−3.19	[R].PSSVNYMVAPVTGNDAGIR.[R]	88–106 aa	1
Neuroglycan C / Chondroitin sulfate proteoglycan 5	*Cspg5*	0.04665	−3.61	[AG].VPAREAGSAIEAEELVR.[S]	31–47 aa	31
Immunoglobulin superfamily member 8	*Igsf8*	0.01245	−2.52	[R].HAAYSVGWEMAPAGAPGPGR.[L]	336–355 aa	28
Alpha‐2 antiplasmin	*Serpinf1*	0.01405	−4.28	[L].AAAVSNFGYDLYR.[L]	55–67 aa	20
V‐type proton ATPase subunit B brain isoform	*Atp6v1b2*	0.01800	−2.26	[G].AAPELPVPTGGPMAGAR.[E]	13–29 aa	1
Protocadherin gamma C3	*Pcdhgc3*	0.02568	−4.51	[A].STIIHYEILEER.[E]	30–41 aa	30
Endoplasmin	*Hsp90b1*	0.01497	−4.36	[A].DDEVDVDGTVEEDLGKSR.[E]	22–39 aa	22
Protein disulfide‐isomerase	*Pdia3*	0.02329	−5.12	[A].SDVLELTDENFESR.[V]	30–43 aa	25
Transmembrane emp24 domain‐containing protein 10	*Tmed10*	0.03895	−2.69	[G].ISFHLPVNSR.[K]	32–41 aa	32
Syntaxin‐7	*Stx7*	0.01538	−3.43	[M].SYTPGIGGDPAQLAQR.[I]	2–17 aa	2
Cell cycle control protein	*Tmem30a*	0.04240	−2.41	[M].AMNYSAKDEVDGGPTGPPGGAAKTR.[R]	2–26 aa	2
Nidogen‐2	*Nid2*	0.04284	−4.29	[A].LRPEELFPYGESWGDR.[L]	31–46 aa	31

^1^ Peptide sequences are shown in single‐letter amino acid (aa) code (N‐terminus to C‐terminus). Dots indicate the boundaries of the identified peptide, and amino acids in brackets denote the flanking residues in the parent protein sequence.

^2^ N‐terminal start positions are based on available annotations and inferred from homologous proteins where necessary.

Analysing the sequences of the identified cleavage fragments showed that the majority corresponded to the N‐termini of the mature proteins (by similarity with human homologues), with fragments of SDCBP and IGSF8 covering internal regions (Table 2). Comparing the detected peptides with the results from a recent N‐terminomic study of proteolytic processing in mouse neurons (Ameen et al., 2023), we found several sequences to overlap either fully or at least in part, supporting that these cleavage events can be observed across independent datasets. Additionally, four fragments (corresponding to CDH13, CLU, HSP90B1, and ATP6V1B2) showed similarity to fragments previously detected in a secretome study of metalloprotease‐dependent ectodomain shedding in human cell lines (Tsumagari et al., 2021) (Table S4).

To further investigate sustained ADAM10 activity on sEVs beyond the substrates identified here by proteomics, we performed complementary experiments using sEVs from mouse neuroblastoma (N2a) cells (Figure S4). As a readout, we focused on shedding of the cellular prion protein (PrP), a GPI‐anchored glycoprotein with multifaceted roles in cell adhesion and neuronal signalling as well as critical relevance in some neurodegenerative diseases. Although PrP was not among the cleavage products detected by our N‐terminal proteomics analysis, it represents an ideal reporter for ADAM10 activity on sEVs, as it is enriched on EVs (Brenna et al., 2020; Falker et al., 2016; Fevrier et al., 2004) and exclusively shed by ADAM10 in the nervous system (Linsenmeier et al., 2018; Song et al., 2024). Following isolation of sEVs (Figure S4A), characterisation by NTA (Figure S4B), and incubation with or without GI for 42 h, sEVs and their supernatants were separated by ultracentrifugation and analysed by immunoblotting (Figure S4C and D). Using a cleavage site‐directed antibody for the specific detection of shed PrP (sPrP) (Linsenmeier et al., 2018) (Figure S4B), we observed strong sPrP signal in the supernatant of untreated sEVs, with the signal considerably reduced upon GI treatment (Figure S4C), suggesting ongoing ADAM10‐mediated PrP shedding on isolated sEVs. The residual sPrP signal in the GI‐treated sample likely reflects incomplete inhibition, potentially from insufficient inhibitor concentration, limited accessibility of vesicle‐associated ADAM10, or pre‐existing cleavage products. In contrast to sPrP, probing for soluble APPα (sAPPα), which is released upon cleavage of full‐length APP by ADAM10 or other α‐secretase activities (notably ADAM17) not inhibited here, showed no noticeable differences between conditions (Figure S4D).

**Figure 6:**
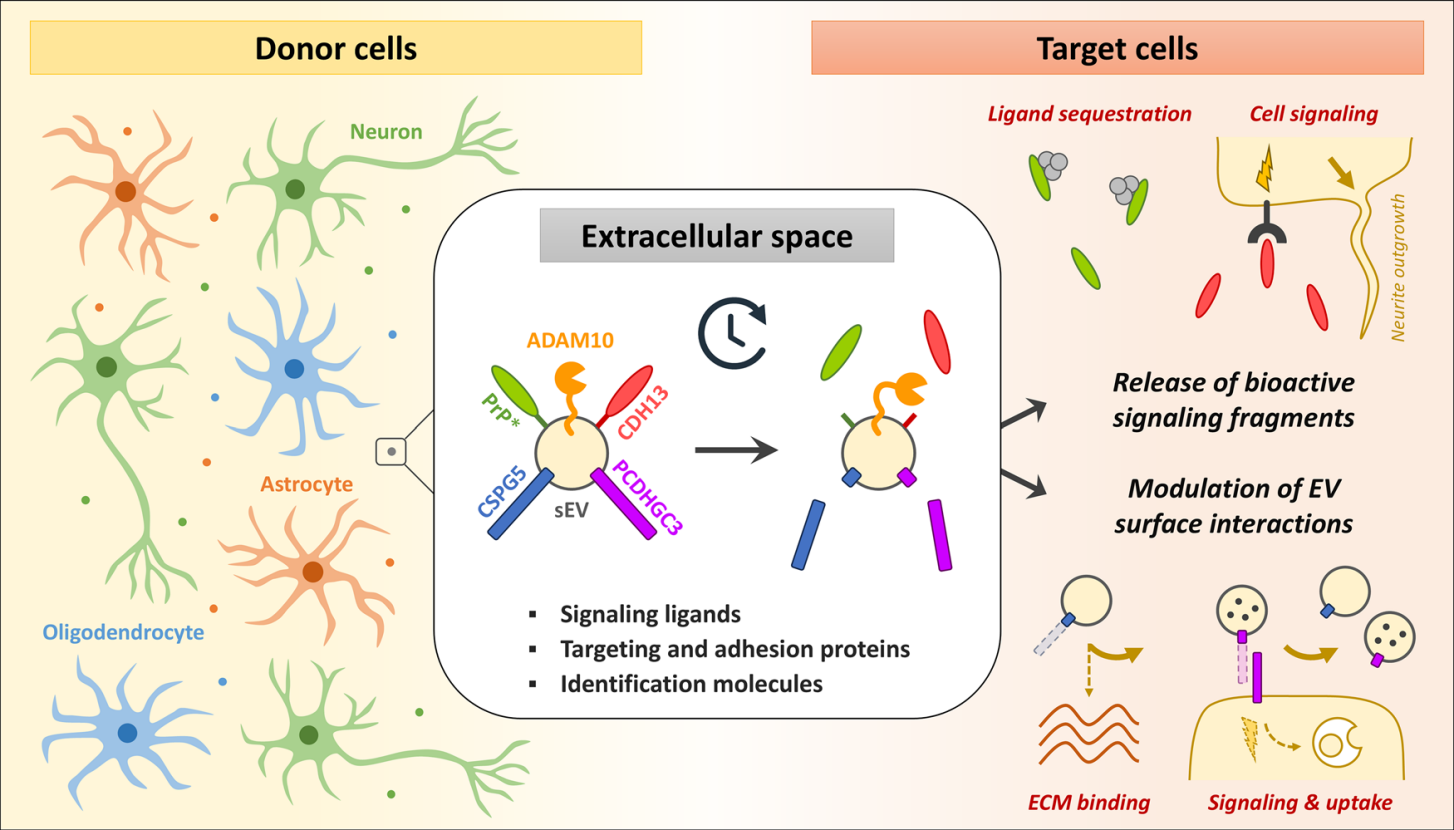
Model of ADAM10‐mediated proteolytic processing on sEVs and potential functional implications. Schematic summary of the study's findings, illustrating ADAM10 substrate candidates identified on sEVs and the proposed functional consequences of their proteolytic processing. Brain cells like neurons, astrocytes, and oligodendrocytes release sEVs carrying active ADAM10 along with surface proteins such as cadherin‐13 (CDH13), neuroglycan C (CSPG5), protocadherin gamma C3 (PCDHGC3), and the prion protein (PrP). Continuous ADAM10 activity on sEVs remodels their surface composition over time—cleaving signalling ligands, adhesion proteins, and identity markers—and may influence EV function *en route* to target cells. On the one hand, ADAM10 activity can release functional fragments into the extracellular space, potentially extending the reach of cell‐derived signals. For example, soluble forms of CDH13, CSPG5, and PrP have been linked to neurite outgrowth and other bioactive effects. Shed PrP may additionally sequester harmful proteopathic oligomers in neurodegenerative diseases. On the other hand, surface cleavage may inactivate ECM‐binding proteins (e.g., CSPG5) or modulate EV‐to‐cell binding (e.g., CDH13, PCDHGC3, PrP), thereby affecting EV mobility, signal transduction, uptake specificity, and cargo delivery dynamics. Notably, microglia and other brain cell types—absent from cortical cultures in this study—likely contribute to EV release. PrP shedding, as indicated by the asterisk, was observed in a complementary experiment using N2a cell‐derived sEVs. Not all ADAM10 substrates necessarily co‐exist on individual sEVs.

In conclusion, N‐terminal proteomics revealed that inhibition of sEV‐ADAM10 activity alters proteolytic processing of a distinct set of membrane‐associated surface proteins involved in cell adhesion, neurite outgrowth, and nervous system development. Based on in‐depth analysis of the relevant literature, many of the identified substrate candidates appear conserved across brain regions and species, supporting the potential relevance of sEV‐ADAM10 activity to human brain physiology. Complementing these findings, targeted experiments in an independent cell model demonstrated continued ADAM10‐mediated shedding of PrP on isolated sEVs, further corroborating the capacity of sEVs to sustain active proteolysis. Taken together, our results suggest sEV‐ADAM10 as a relevant modulator of EV composition and neuronal function, with its continuous activity towards surface proteins likely impacting EV‐cell interactions and contributing to the release of bioactive mediators (Figure 6).

## 4 Discussion

Continuous activity of EV‐resident proteases has the potential to be a vital modulator of EV biology, with a given EV's effects and signalling capacity being dynamically changed over time rather than being predetermined in the moment of EV secretion. In this study, we focused on ADAM10 as a widely expressed sheddase with important regulatory functions, and investigated its role on brain cell‐derived sEVs. We found that neuronal and synaptic processes associated with sEVs, as identified by GO analysis, involve several proteins that are known cellular ADAM10 substrates, suggesting the possibility that ADAM10 could modulate EV function through processing of its substrates. The key finding of this work is that sEV‐ADAM10 is proteolytically active on sEVs from cortical cultures and N2a cells, and that its inhibition affects membrane‐bound sEV proteins involved in processes like cell adhesion, vesicle trafficking, neurite outgrowth, and nervous system development. Elucidating the biological consequences will require further studies for each substrate candidate; however, this discovery points to a mechanism that could influence neuronal function, modulate EV‐cell delivery, and enable the release of soluble signalling fragments over time.

It is generally accepted that ADAM10 is active on EVs from non‐neuronal cells (Mathews et al., 2010; Preußer et al., 2026; Stoeck et al., 2006; Tosetti et al., 2018; Yoneyama et al., 2018), and here we confirmed its activity on sEVs isolated from primary rat cortical cultures using a fluorogenic ADAM10‐specific peptide substrate. In contrast to other studies, sEV‐ADAM10 activity in our hands showed a slightly different profile over 3 h, with a more linear signal increase in comparison to Hansen et al. (2016) using EVs derived from a Hodgkin lymphoma cell line, and a relatively low activity compared to Lee et al. (2022) using plasma‐derived EVs in combination with an activity assay kit for α‐secretases (including the major α‐secretase ADAM10 (Jorissen et al., 2010; Kuhn et al., 2010)). Several factors, including the number of assayed EVs and differences in EV isolation protocols and buffers, may contribute to the variation in the described activity patterns. The cellular origin of the EVs, which primarily determines the membrane composition of the secreted vesicles, plays another key role as ADAM10's surface abundance, stability, and activity are regulated by many interactors such as tetraspanins, tissue inhibitors of metalloproteinases (TIMPs), lipids, and other proteases (Saftig and Lichtenthaler, 2015; Seipold et al., 2018; Tosetti et al., 2021). Although peptide substrate assays can vary in specificity, the fluorogenic peptide employed in our study has been characterised as highly selective for ADAM10 (Caescu et al., 2009). In addition, this substrate contains a charged tail that prevents diffusion through lipid membranes. As ADAM10 is known to have the same membrane orientation on EVs as on cells (with its N‐terminal catalytic domain facing the extracellular space as a structural prerequisite for its proteolytic activity) (Cvjetkovic et al., 2016; Groth et al., 2016), the recorded ADAM10 activity occurred on the surface of sEVs as opposed to lumen‐facing ADAM10 from intracellular (i.e., secretory or endocytic) vesicles.

To study sEV‐ADAM10 activity towards sEV membrane proteins, previous reports focused on specific ADAM10 substrates, either by analysing the soluble N‐terminal fragment of L1CAM in the cell supernatant (Gutwein et al., 2003; Stoeck et al., 2006) or by detecting C‐terminal fragments of APP remaining in the EV membrane (Lauritzen et al., 2019; Pérez‐González et al., 2020; Sharples et al., 2008). In the present work, we instead used N‐terminal proteomics as a novel tool for an untargeted screening of EV‐based proteolytic events. We identified a panel of sixteen sEV‐ADAM10 substrate candidates to be significantly altered upon pharmacological sEV‐ADAM10 inhibition. Among them, the cell adhesion molecule PCDHGC3 has previously been characterised as an ADAM10 substrate in mouse embryos and neuronal cells, with ADAM10 acting as the major protease mediating its shedding and thereby modulating cell‐cell adhesion (Reiss et al., 2006). In addition, a comprehensive proteomic study comparing the secretome of conditional ADAM10 knockout primary neurons with their respective wild‐type samples identified CDH13, CLU, and CSPG5 among proteins that were significantly reduced (Kuhn et al., 2016). The remaining twelve proteins represent novel ADAM10 substrate candidates and may even reflect processing events specific to the sEV membrane, which warrants further investigation. While N‐terminal proteomics proved valuable in identifying changes in membrane‐associated EV proteins, we also observed unexpected alterations in intraluminal cargo, which could reflect indirect or secondary effects, or some degree of contamination. Future studies could minimise potential contamination with intracellular proteins by incorporating additional purification steps, such as density gradient centrifugation or size‐exclusion chromatography.

Due to the nature of the sample preparation for N‐terminal proteomics (Figure 4A), and considering that information about protease‐specific cleavage sites is limited, it is difficult to infer ADAM10 cleavage sites from the identified N‐terminal fragments. ADAM10‐mediated shedding typically occurs close to the substrate's transmembrane domain and thereby releases the protein's ectodomain (Lipper et al., 2023), a fragment that—after trypsin digestion for mass spectrometry—will only be identifiable by its most N‐terminal sequence. Consistent with this, many of the identified fragments correspond to the most N‐terminal site of the mature protein. Moreover, all sequences contain an arginine (R) at the P1 cleavage site position (Table 2), which represents a common motif for trypsin cleavage (Olsen et al., 2004). On the other hand, arginine at the P1 position has also been suggested to be a preferred residue for ADAM10‐mediated cleavage (Chen et al., 2014), and several of our detected fragment sequences have been identified in previous proteomic studies of proteolytic processing and ectodomain shedding (Table S4) (Ameen et al., 2023; Tsumagari et al., 2021). Further investigations, for example complementary C‐terminal proteomic analysis and cell biological assessment, will be required to validate the putative substrates and determine their exact cleavage sites.

While we generally expect ADAM10‐mediated shedding to occur on the same membrane in *cis* (Figure 6), which is the most established mechanism (Lichtenthaler et al., 2018), we cannot exclude proteolytic interactions in *trans* between vesicles. Since the distribution of ADAM10 and its substrates may vary between individual EVs and between EV populations from different cell sources, changes in EV composition and secretion—for example under stress—could theoretically influence substrate accessibility and the local generation of cleavage products. Moreover, preferential enrichment of ADAM10 on EVs derived from distinct cell types (e.g., neuronal versus glial populations) could point to origin‐specific rather than ubiquitous functions. Resolving the precise co‐localisation of protease and substrates, as well as their distribution across EV subtypes, will require cell type‐specific isolation and single‐EV profiling approaches (e.g., surface marker‐based sorting) in future work.

Although the inhibitor GI is widely recognised as ADAM10‐selective, some degree of cross‐reactivity with other proteases, such as ADAM17 or matrix metalloproteinases, cannot be fully excluded. Nonetheless, several factors support ADAM10 as the primary contributor to the observed effects on EV membrane proteins: *(i)* GI exhibits over 100‐fold greater potency against ADAM10 compared with ADAM17 (Ludwig et al., 2005), and a recent study confirmed that while GI completely abolished ADAM10 activity, autocatalytic processing of ADAM17 was unaffected by this treatment (Song et al., 2024); *(ii)* the performed experiments were conducted under constitutive rather than stimulated/induced conditions, typically favouring ADAM10 over ADAM17 activity; and *(iii)* GI treatment led to a significant reduction of PCDHGC3, for which ADAM10 is the major sheddase on the cell surface (Reiss et al., 2006). While genetic models are frequently used to support pharmacological findings, their value for insights into proteolysis on EVs is limited, since modulation of ADAM10 in donor cells—including constitutive or conditional gene knockout and catalytic mutants—can already alter protein sorting, EV composition, and possibly EV production per se (Mathews et al., 2010; Seifert et al., 2020; Wichert et al., 2017). Especially changes to the latter are to be expected, as recent findings described a relevant role of cell surface ADAM10 in the detachment and release of sEVs from donor cells (Bizingre et al., 2025). Pharmacological inhibition of isolated sEVs therefore offered a more specific approach to reveal proteolytic differences between treated and untreated EVs derived from the very same pool of otherwise unaltered vesicles. To further establish the biological relevance of sEV‐ADAM10 activity, future investigations should directly compare GI‐inhibited and untreated sEVs in functional assays, for example assessing effects on neurite outgrowth, cell adhesion, neuroprotection, or EV uptake in recipient cells. In parallel, the development of complementary strategies (including genetic candidate depletion) will enable more precise dissection of proteolytic events on EVs and their downstream biological consequences.

We can expect that the list of physiological sEV‐ADAM10 substrates is even larger and presumably tissue‐ and context‐dependent. For example, none of the previously reported substrates such as L1CAM and CD44 were significantly affected by sEV‐ADAM10 inhibition, which could be due to the use of primary rat cortical cell cultures as a different source of EVs or differences in EV isolation protocols. More surprisingly, apart from PCDHGC3, none of the other prominent cellular ADAM10 substrates present in our sEV preparations (e.g., NOTCH1, APP, N‐cadherin, and NCAM1) (Table 1) were detected to be affected by sEV‐ADAM10 inhibition. Since our complementary candidate approach using sEVs from N2a cells supported continued ADAM10‐mediated release of sPrP but not sAPPα from sEVs (Figure S4), this may point to a complex regulation network on the surface of EVs favouring shedding of certain substrates over others. This, in turn, might be modulated by the differential presence of relevant interactors of both, protease and substrate, and should be studied in the future. Additional biochemical confirmation of further canonical and emerging ADAM10 substrates, for example using fragment‐specific antibodies, will also be valuable to extend these findings.

Notably, as we could not utilise PIs for EV isolation (as this would have impacted ADAM10 activity readouts), several proteins may have already been processed (or even partially degraded) before the start of the experiment. Although proteases, not just EV‐associated but also soluble ones present in the culture supernatant, are a common adversary to isolating full‐length proteins of interest, many EV studies either *(i)* do not use PIs, *(ii)* do not report the use of PIs, or *(iii)* use general PIs yet without EDTA, hence leaving certain metalloproteases such as ADAM10 partially active (Brummer et al., 2018). This could lead to unreproducible results or inaccurate interpretations when targeting EV surface proteins for biochemical and cellular studies, or when using antibody‐based methods to isolate EVs for biomarker discovery. Notably, since the *Minimal Information for Studies of Extracellular Vesicles* (MISEV) guidelines of 2018 (Théry et al., 2018) and 2023 (Welsh et al., 2024) as well as a ‘Membranes and EVs’ workshop position paper by the *International Society for Extracellular Vesicles* (ISEV) (Russell et al., 2019) are lacking such recommendations thus far, the present findings may serve as an example and additional impetus to consider the impact of protease activity in EV preparations, advising the use of effective and broad‐range PIs for EV preparations whenever applicable.

Continuous cleavage and ‘deactivation’ of surface recognition molecules on sEVs may further influence EV‐cell interactions. Notably, the panel of ADAM10‐regulated proteins we identified here includes several cell adhesion molecules and proteins with established binding functions. Among these, CDH13 and PCDHGC3 were previously attributed with EV‐cell interaction properties (Wang et al., 2020). In addition, we observed sEV‐based ADAM10‐mediated shedding of PrP, a neuronal cell adhesion and signalling protein with diverse suggested roles on EVs (Brenna et al., 2020; D'Arrigo et al., 2021; Gonias et al., 2022). For example, EVs derived from PrP‐deficient mouse brain are more rapidly endocytosed by recipient neurons and sorted to lysosomes, whereas PrP‐carrying EVs exhibit slower uptake and may undergo fusion at the plasma membrane (Brenna et al., 2020). These findings suggest that cleavage of PrP and related surface proteins may modulate not only the specificity of EV targeting but also the uptake route, kinetics, and eventual functional outcomes in recipient cells. Furthermore, proteolytic processing of extracellular matrix (ECM)‐binding proteins such as CSPG5 could affect EV mobility and spatial distribution within the extracellular space. Similarly, cleavage of ECM structures (e.g., basement membranes) or other ‘barriers’ by EV surface proteases (like ADAM10) may contribute to EV passage across tissues. Together, these observations support a model in which the molecular ‘delivery code’ embedded on EV surfaces is subject to dynamic remodelling *en route* to recipient cells, driven by sustained proteolytic activity (Figure 6).

In addition to influencing direct EV‐to‐cell and EV‐to‐matrix binding, ongoing ADAM10 activity may liberate bioactive cleavage fragments (with possibly low intrinsic biostability and hence relatively transient effectivity) from the EV surface over time in a more localised manner. In this line of thought, EVs could be regarded as ‘space shuttles’ ensuring protected long distance cargo transport, with ADAM10 being the engineer working on the exterior. Not only would its activity change navigation and recognition sites, but it may also release functional fragments into the extracellular space, which can then signal more locally within the target environment (Mohammadi et al., 2023). This way, EVs would extend the range and area of effect beyond the half‐life of the metabolites otherwise conventionally released from the secreting donor cell. Although currently speculative and experimentally challenging, this concept deserves closer examination.

Remarkably, some of the detected proteins affected by sEV‐ADAM10 activity are linked to a reported functionality of their cleaved soluble fragments. The proteolytically released ectodomain of neuroglycan C (CSPG5) has been suggested to promote neurite outgrowth during brain development (Shuo et al., 2007; Nakanishi et al., 2006), while a recombinant ectodomain of T‐cadherin (CDH13) has been shown to inhibit neurite outgrowth from motor neurons in vitro (Fredette et al., 1996). Beyond the nervous system, soluble CDH13 has also been implicated in metabolic regulation, improving glucose homeostasis and β‐cell proliferation in a murine model of diabetes and obesity (Okita et al., 2022). Notably, the same group identified three distinct soluble CDH13 isoforms in human serum, associated with clinical parameters in type 2 diabetes patients, and speculated that these may originate from EVs, although the responsible protease remained unidentified thus far (Fukuda et al., 2021).

Additionally, sPrP has been linked to diverse physiological roles, including neurite outgrowth, immune modulation, tumour progression, and cancer drug resistance (Amin et al., 2016; Kanaani et al., 2005; Mantuano et al., 2022; Provenzano et al., 2017; Wiegmans et al., 2019). Importantly, sPrP is thought to exert neuroprotective effects by sequestering and blocking extracellular toxic protein assemblies, such as amyloid‐β (Fluharty et al., 2013; Linsenmeier et al., 2021; Mohammadi et al., 2023; Nieznanski et al., 2012; Song et al., 2024). Given that EVs have also been implicated in the spread of misfolded, infectious PrP species in prion disease models (Fevrier et al., 2004; Khadka et al., 2023; Vella et al., 2007), it remains to be determined whether ADAM10 can also shed misfolded PrP from EVs.

In sum, our findings support a model in which active proteolysis on EV surfaces generates potent soluble mediators capable of acting in an autocrine, paracrine, or endocrine manner. ADAM10 emerges as a key protease in this context, underscoring the need for further investigations into the functional implications of EV‐based shedding mechanisms (Figure 6).

Finally, we observed that ADAM10 remains active on sEVs from cortical cell cultures over an extended period in vitro and that the isolated sEVs remain stable for at least 24 h, providing insight into the time frame during which EV‐associated proteolysis may occur. In vivo studies in mice have shown half‐lives of EVs in the circulation ranging from minutes to a few hours, although EV‐associated signals can still be detected in tissues several hours after administration (Kang et al., 2021; Lai et al., 2014). These observations indicate that EVs stay intact long enough to exert functional effects, suggesting that ongoing ADAM10‐mediated shedding at the EV surface may contribute to gradual membrane remodelling and the release of soluble fragments in a physiologically and pathologically relevant manner. In addition, since the presence of the novel sEV‐ADAM10 substrate candidates identified here appears conserved between species, it is likely that the proteolytic capacity observed for rodent sEV‐associated ADAM10 extends to EVs derived from the human brain. Considering the significance of ADAM10 in health and disease, its potential regulatory role on EVs may present a missing link to aberrant EV signalling in diseases associated with dysregulated ADAM10 activity such as neurodegeneration, inflammation, and cancer.

## 5 Conclusions

Our study highlights brain cell‐derived EVs as platforms for continued proteolytic processing and suggests sEV‐ADAM10 as a potential modulator of EV fate and function by reshaping the surface protein composition after release from the donor cell. While we did not observe effects of sEV‐ADAM10 inhibition on some known prominent substrates, we found novel substrate candidates and established a conceptual and methodological foundation for future investigations into EV‐based proteolytic activity. Given ADAM10's relatively ubiquitous tissue expression, constitutive activity, and consistent presence on EVs from various cell types, it likely plays a critical role in the temporal regulation of EV trafficking and may extend to other systems and pathologies, including immune modulation and cancer. Our study also warrants consideration of proper protease inhibition in respective EV studies, especially when focusing on surface protein biomarkers or substrate candidates, or when investigating molecular interactions between EVs and target cells. Further studies are needed to clarify the functional significance of sEV‐ADAM10 in the brain and in other contexts to better understand the roles of EV‐resident proteases in health and disease.

## Author Contributions

**Christopher C. Reimann**: conceptualization; formal analysis; investigation; methodology; visualization; writing – original draft; writing – review and editing. **Hermann C. Altmeppen**: formal analysis; investigation; writing – review and editing. **Tomas Koudelka**: Formal analysis; investigation; writing – review and editing. **Michaela Schweizer**: Formal analysis; investigation; writing – review and editing. **Andreas Tholey**: Formal analysis; investigation; writing – review and editing. **Behnam Mohammadi**: formal analysis; investigation; methodology. **Julia Bär**: Formal analysis; investigation; writing – review and editing. **Lesley Cheng**: Conceptualization; formal analysis; funding acquisition; investigation; project administration; supervision; writing – original draft; writing – review and editing. **Markus Glatzel**: Conceptualization; formal analysis; investigation; project administration; resources; supervision; writing – original draft; writing – review and editing. **Marina Mikhaylova**: Conceptualization; formal analysis; funding acquisition; investigation; project administration; resources; supervision; writing – original draft; writing – review and editing. **Andrew F. Hill**: conceptualization; formal analysis; funding acquisition; investigation; project administration; resources; supervision; writing – original draft; writing – review and editing.

## Conflicts of Interest

Andrew F. Hill and Lesley Cheng are co‐founders, shareholders, and directors of Excelligent Pty Ltd, an academic spin‐out company that develops diagnostics for neurological diseases. Andrew F. Hill is Editor in Chief of the Journal of Extracellular Biology. The submission was handled independently by another editor to ensure the integrity of the peer‐review process, and Andrew F. Hill was not involved in the editorial decision.

## Supporting information




**Supporting information**: Supplementary Figures S1‐S4.

**Supporting Information: Table S1**: Proteomics dataset of identified proteins from CMFs and sEVs.

**Supporting Information: Table S2**: Gene Ontology analysis of CMF and sEV proteomes and of known ADAM10 substrates present in the sEV samples.

**Supporting Information: Table S3**: Comparison of EV proteomics datasets from published reports.

**Supporting Information: Table S4**: N‐terminal proteomics dataset of identified proteins from 0 h, 24 h, and 24 h + GI sEVs.

## Data Availability

The data that supports the findings of this study are available in the supplementary material of this article
