# Supplementary material for: ADAM10‐Mediated Proteolytic Remodelling of Signalling and Adhesion Proteins on Brain Cell‐Derived Small Extracellular Vesicles
Source: J Extracell Biol. 2026 Apr 17;5(4):e70129. doi: 10.1002/jex2.70129 (PMC13088879; doi:10.1002/jex2.70129)
Supplement: Supplementary file 1 — Supporting information: Supplementary Figures S1‐S4. [file JEX2-5-e70129-s002.docx]

# Supplementary Figures


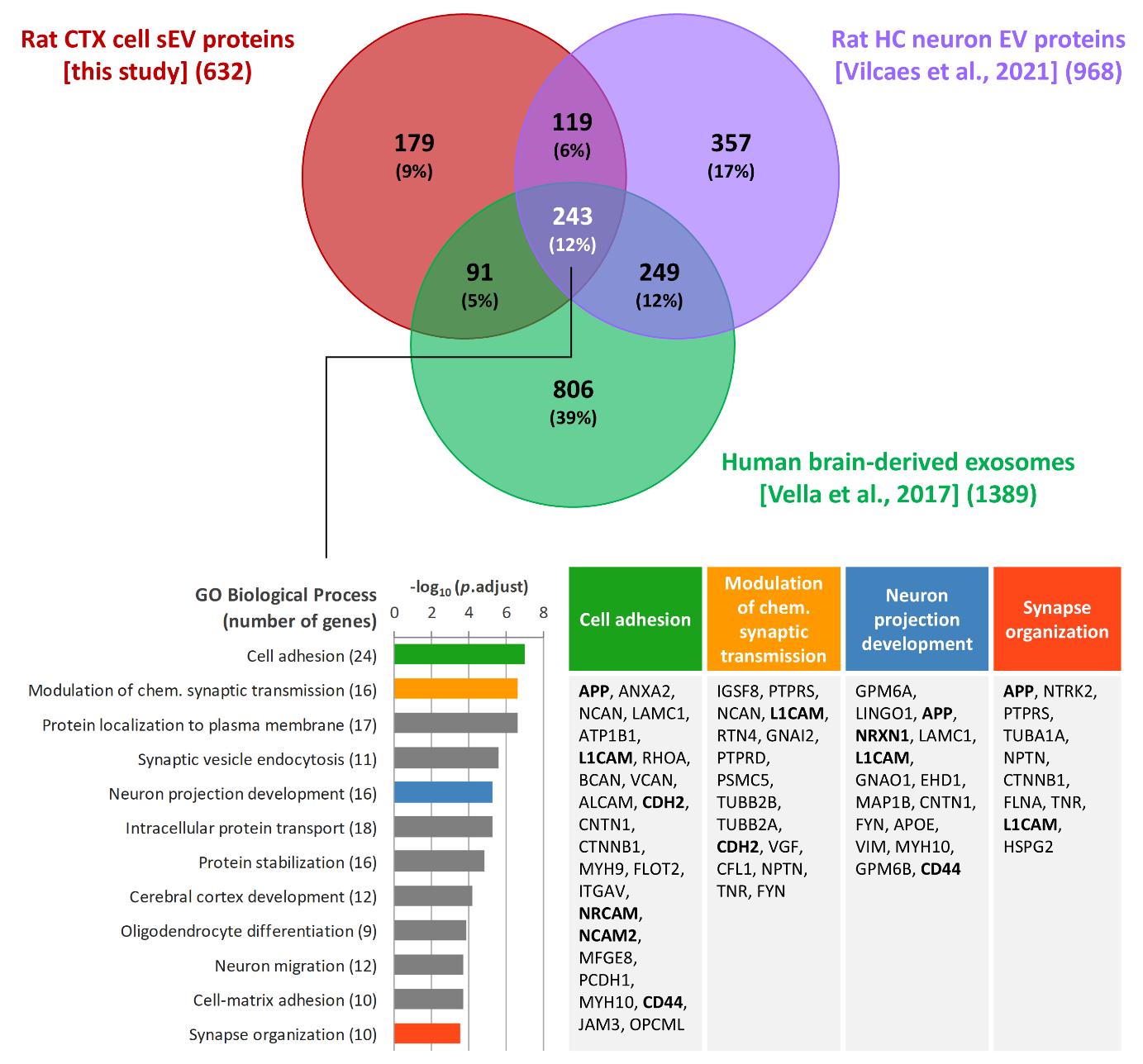


Supplementary Figure S1. EV proteome comparison showing that EV-related biological processes and correlating ADAM10 substrates in rat cortical culture-derived sEV proteome are conserved between brain regions and species.

Proteome comparison of our sEVs from cultured DIV13-15 primary rat cortical (CTX) cells with EVs from cultured primary rat hippocampus (HC) neurons (Vilcaes et al., 2021) and human brain-derived exosomes (Vella et al., 2017). The 243 proteins identified in all three datasets were analyzed for Gene Ontology (GO) biological process terms using DAVID. Selected GO terms with the most significant adjusted *p*-values (Benjamini correction) are plotted against their -log_10_ (*p*.adjust). Proteins involved in selected processes are shown in the table. Protein names in bold indicate known cellular ADAM10 substrates.

**
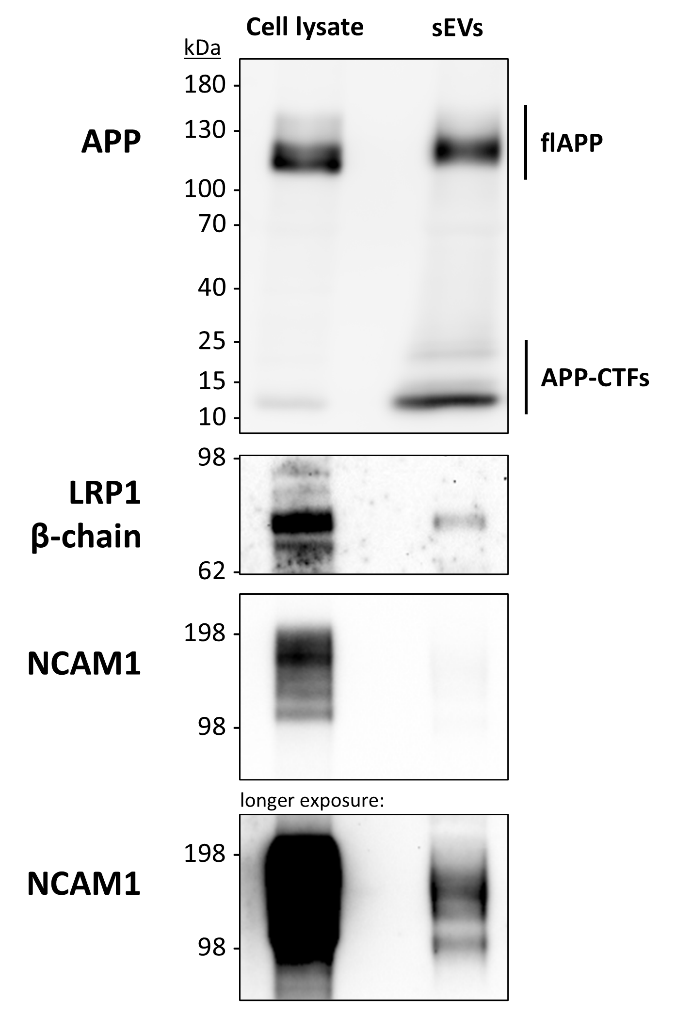
**

Supplementary Figure S2. Presence of known cell surface ADAM10 substrates in sEV samples from cortical cultures.

Immunoblot validation of selected known ADAM10 substrates identified in sEV samples by mass spectrometry (Table 1). flAPP = full-length APP; APP-CTFs = APP C-terminal fragments.


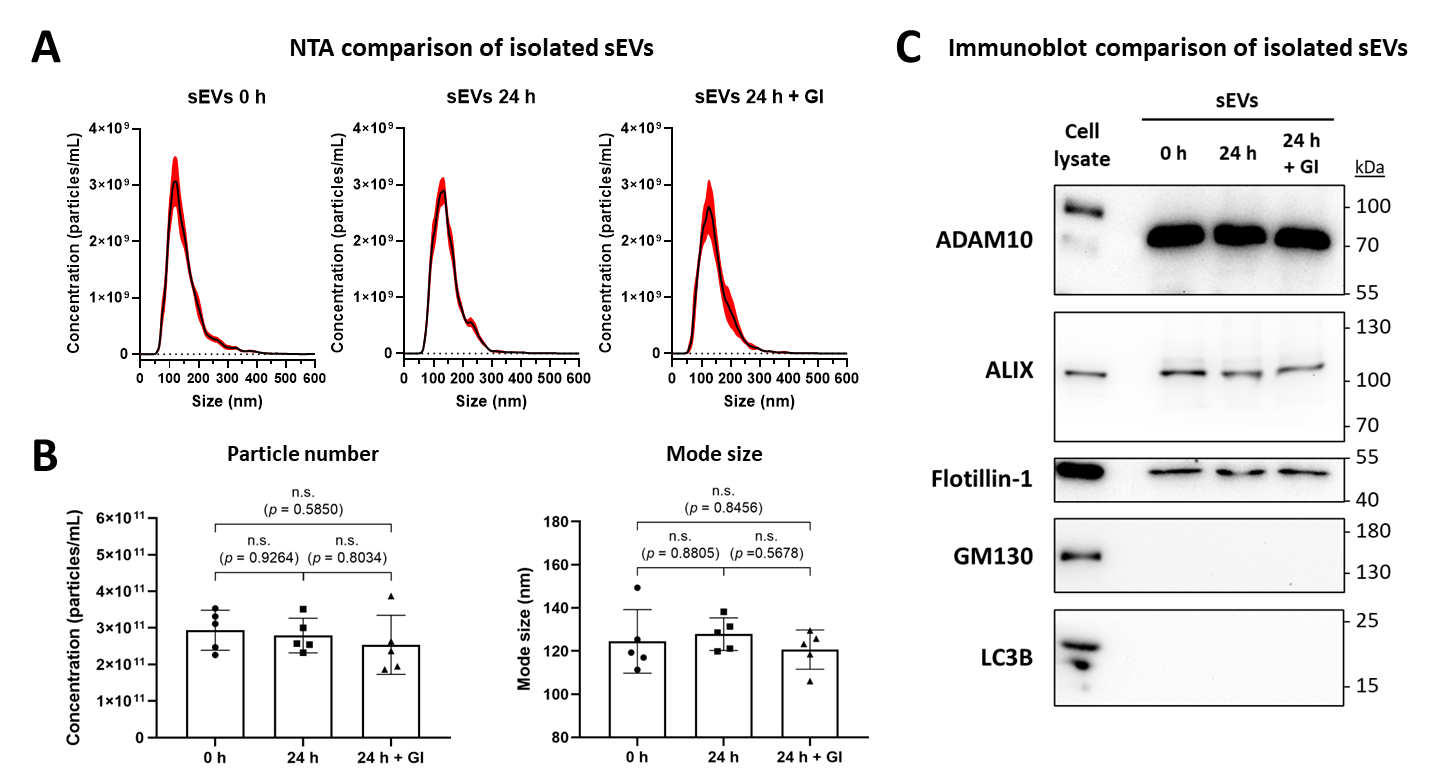


Supplementary Figure S3. Cortical culture-derived sEVs are stable over 24 h at 37 °C.

Isolated cortical culture-derived sEVs were either used freshly prepared (0 h) or incubated at 37 °C with or without ADAM10 inhibitor GI254023X (GI) in 20 mM HEPES buffer. For particle analysis by NTA, samples from 0 h sEVs were taken and measured directly after resuspension, and 24 h samples were taken directly from the upper part of the sEV suspension without mixing. No significant differences in size profiles (**A**), particle numbers and mode sizes (**B**) were detected between groups. n = 5 with SEM, ordinary one-way ANOVA (Tukey’s multiple comparisons test, unpaired), n.s. (non-significant, *p* > 0.05). (**C**) Representative immunoblots of isolated sEVs (and a cell lysate on the left as control), probing for EV positive markers ADAM10 (C-terminal antibody, Abcam, Cat# ab124695), ALIX, and flotillin-1, and EV negative markers GM130 and LC3B. Note that the upper ADAM10 band in the cell lysate corresponds to the inactive pro-ADAM10 form (not sorted to EVs).


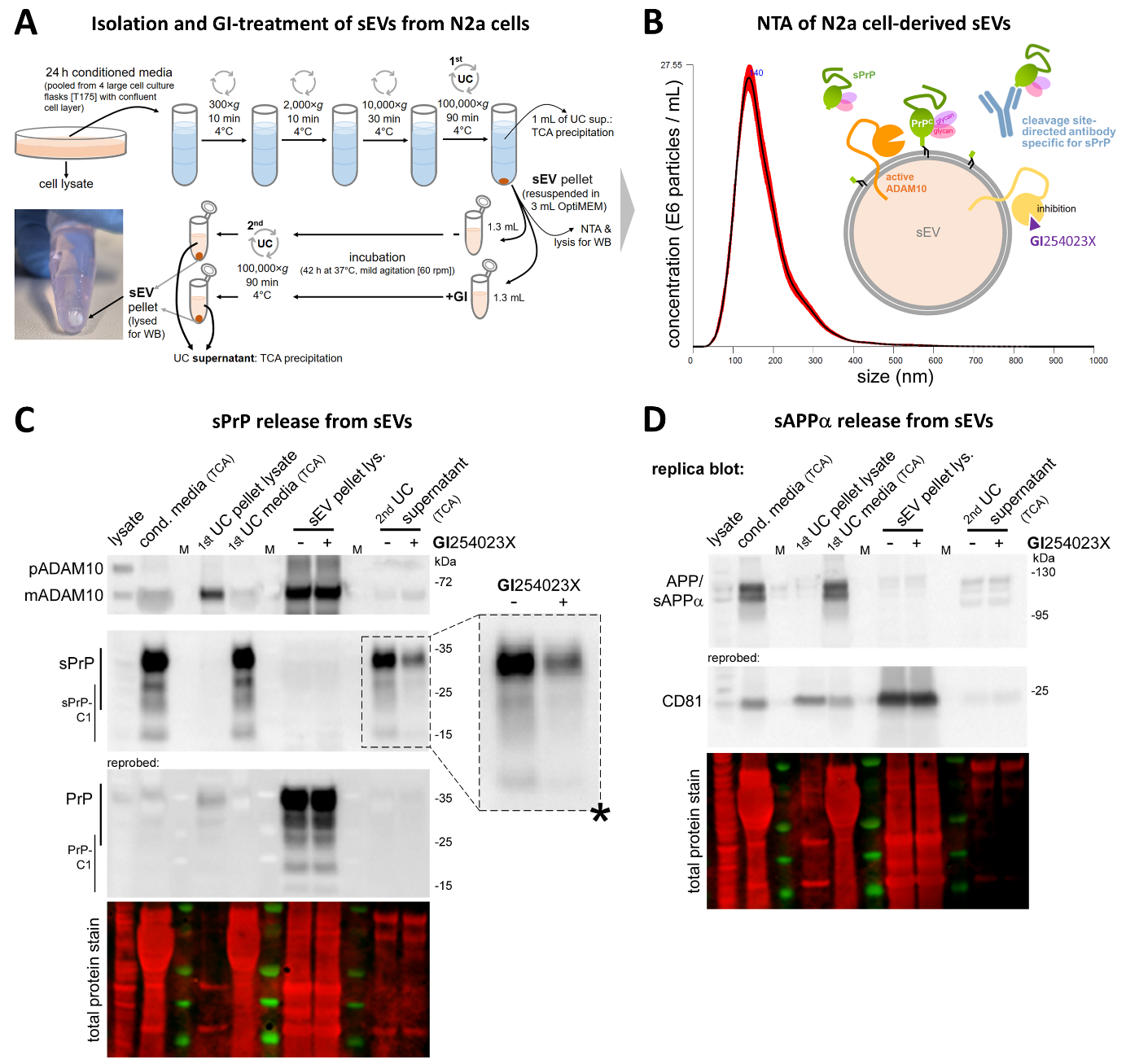


**Supplementary Figure S4. ADAM10-mediated shedding of PrP continues on the surface of isolated sEVs.**

sEVs were isolated from mouse neuroblastoma (N2a) cells (A), characterized by NTA (B), and analyzed by immunoblotting (C-D) as previously described in Linsenmeier et al. (2018), with slight modifications depicted herein. (**A**) N2a cells were conditioned in Opti-MEM for 24 h and the conditioned media was differentially centrifuged and finally ultracentrifuged (UC) in polypropylene tubes (Beckman Coulter). The sEV pellet was resuspended in Opti-MEM, split into two equal volumes, and incubated with or without 30 µM of GI for 42 h at 37 °C. Following a second UC step, the sEV pellet was lysed in a 1:1 mixture of 4× sample buffer with RIPA buffer for western blot (WB), and the separated sEV supernatant was precipitated with trichloroacetic acid (TCA) to concentrate all protein fragments. Additional samples for WB were collected at different steps of the procedure. (**B**) NTA was performed with the initial pool of sEVs and demonstrated a high yield of particles (3.2 × 10^12^ ± 1.7 × 10^11^ particles/mL) with a mode size of 138 ± 3 nm. The scheme illustrates the subsequent incubation of the sEVs with or without GI and detection of released PrP shedding fragments (sPrP) using a highly sensitive and cleavage site-specific antibody (sPrP^G227^, Linsenmeier et al., 2018). (**C**) Western blot analysis of collected samples probing for mature (m) and pro-ADAM10 (p) forms (clone EPR5622, C-terminal, Abcam, Cat# ab124695), sPrP (including its N-terminally truncated shed C1 fragment resulting from PrP α-cleavage), and membrane-anchored full-length PrP and its C1 fragment (clone POM1, Merck, Cat# MABN2285). The banding pattern of PrP is due to the presence of di-, mono-, and non-glycosylated forms. Total protein staining confirms equal sample loading for 2^nd^ UC sEV pellets and supernatants after experimental incubation (on the right half of the blot). The asterisk-marked box highlights the difference in sPrP levels in the absence or presence of GI. Of note, the residual sPrP signal in the GI-treated sample likely reflects pre-existing cleavage products or incomplete inhibition due to insufficient inhibitor concentration and/or limited accessibility of all ADAM10 molecules. (**D**) Replica blot from the same experiment detected with antibodies against APP/sAPPα (clone Poly18058, BioLegend, Cat# 805801) and EV marker CD81 (clone D3N2D, Cell Signaling, Cat# 56039).

# References

Linsenmeier, L., Mohammadi, B., Wetzel, S., Puig, B., Jackson, W. S., Hartmann, A., Uchiyama, K., Sakaguchi, S., Endres, K., Tatzelt, J., Saftig, P., Glatzel, M., & Altmeppen, H. C. (2018). Structural and mechanistic aspects influencing the ADAM10-mediated shedding of the prion protein. *Molecular Neurodegeneration*, *13*(1), 1–17. https://doi.org/10.1186/s13024-018-0248-6

Vella, L. J., Scicluna, B. J., Cheng, L., Bawden, E. G., Masters, C. L., Ang, C. S., Willamson, N., McLean, C., Barnham, K. J., & Hill, A. F. (2017). A rigorous method to enrich for exosomes from brain tissue. *Journal of Extracellular Vesicles*, *6*(1). https://doi.org/10.1080/20013078.2017.1348885

Vilcaes, A. A., Chanaday, N. L., & Kavalali, E. T. (2021). Interneuronal exchange and functional integration of synaptobrevin via extracellular vesicles. *Neuron*, *109*(6), 971-983.e5. https://doi.org/10.1016/j.neuron.2021.01.007
